# Insulin deprivation induces PP2A inhibition and tau hyperphosphorylation in hTau mice, a model of Alzheimer’s disease-like tau pathology

**DOI:** 10.1038/srep46359

**Published:** 2017-04-12

**Authors:** Maud Gratuze, Jacinthe Julien, Franck R. Petry, Françoise Morin, Emmanuel Planel

**Affiliations:** 1 Université Laval, Faculté de médecine, Département de Psychiatrie et Neurosciences, Québec, QC, Canada; 2 Centre de Recherche du CHU de Québec, Axe Neurosciences, Québec, QC, Canada

## Abstract

Abnormally hyperphosphorylated tau aggregated as intraneuronal neurofibrillary tangles is one of the two neuropathological hallmarks of Alzheimer’s disease (AD). The majority of AD cases are sporadic with numerous environmental, biological and genetic risks factors. Interestingly, insulin dysfunction and hyperglycaemia are both risk factors for sporadic AD. However, how hyperglycaemia and insulin dysfunction affect tau pathology, is not well understood. In this study, we examined the effects of insulin deficiency on tau pathology in transgenic hTau mice by injecting different doses of streptozotocin (STZ), a toxin that destroys insulin-producing cells in the pancreas. One high dose of STZ resulted in marked diabetes, and five low doses led to a milder diabetes. Both groups exhibited brain tau hyperphosphorylation but no increased aggregation. Tau hyperphosphorylation correlated with inhibition of Protein Phosphatase 2A (PP2A), the main tau phosphatase. Interestingly, insulin injection 30 minutes before sacrifice partially restored tau phosphorylation to control levels in both STZ-injected groups. Our results confirm a link between insulin homeostasis and tau phosphorylation, which could explain, at least in part, a higher incidence of AD in diabetic patients.

Alzheimer’s disease (AD) is the most widespread neurodegenerative disease affecting mainly elderly. Sporadic, late onset AD is the most common (99%), while genetic forms occurring earlier in life represent less than 1% of AD patients. The exact causes for sporadic AD are not fully understood but epidemiological studies have identified numerous environmental, biological and genetic risks factors, with aging being the most important^1^. Interestingly, there is increasing evidence suggesting that insulin dysfunction is an important risk factor for sporadic AD^2^,^3^. Studies have reported altered glucose metabolism and abnormalities in insulin and insulin receptors levels in brains of AD patients^4^,^5^,^6^,^7^,^8^, and a higher incidence of AD has been detected in patients with diabetes mellitus (DM)^9^,^10^,^11^,^12^. Moreover, the 2014 Alzheimer’s Association report^13^ reported 29% of co-morbidity between DM and AD.

Abnormally hyperphosphorylated tau aggregated as intraneuronal neurofibrillary tangles is one of the two neuropathological hallmarks of AD^14^ along with extracellular accumulation of senile plaques composed of amyloid-β peptide (Aβ)^15^. Numerous studies have demonstrated that insulin is able to modulate tau phosphorylation both *in vitro* and *in vivo* (see ref. 16 for review). This impact of insulin on tau could explain, at least in part, co-morbidity between DM and AD since tau hyperphosphorylation has been shown to induce tau pathology^17^, which correlates with the degree of cognitive impairment in Alzheimer’s disease^18^,^19^,^20^,^21^.

Previous studies have evaluated tau phosphorylation in wild type rodents with insulin-dependent DM induced by intra-peritoneal injection of streptozotocin toxin (STZ)^9^,^22^,^23^,^24^,^25^. However, the mechanisms underlying this hyperphosphorylation are controversial; some studies have implicated Glycogen Synthase Kinase-3β (GSK-3β activation^24^, while others Protein Phosphatase 2A (PP2A) inhibition^22^,^23^ or both^25^. These differences in mechanisms might come from the doses of STZ used. Interestingly, whatever the dose of STZ used, insulin injection restores physiological tau phosphorylation in WT mice^22^,^23^, but it is unknown whether it could rescue tau pathology.

The goals of our study were: (i) to determine whether a single high dose or multiple low doses of STZ induce tau hyperphosphorylation by different mechanisms; and (ii) to determine whether insulin can rescue both tau hyperphosphorylation and pathology in a mouse model of tauopathy.

Therefore, we examined the effects of insulin deficiency on tau pathology in transgenic hTau mice, expressing human tau protein without mutations (tau mutations cause FTDP-17, not AD). We compared two protocol of Type 1 DM (T1DM) induction: one high dose of STZ resulting in marked diabetes (STZ HD group) or 5 low doses leading to a milder T1DM (STZ LD group). Both groups exhibited tau hyperphosphorylation without aggregation in the brain. Tau hyperphosphorylation correlated with inhibition of PP2A, the main tau phosphatase. Some mice exhibited more widespread tau hyperphosphorylation due to diabetes-induced hypothermia. Interestingly, insulin injection 30 minutes before sacrifice partially restored physiological tau phosphorylation to control levels in both STZ-injected groups. Our results confirmed a link between insulin homeostasis and tau phosphorylation, which could, at least, contribute to co-morbidity between diabetes and AD.

## Results

Reminders (Fig. 1)

**STZ LD group:** mice injected with 5 low doses of STZ

**STZ HD group:** mice injected with 1 high dose of STZ

**STZ LD Ins group:** mice injected with 5 low doses of STZ + 1 injection of insulin 30 min before sacrifice

**STZ HD Ins group:** mice injected with 1 high dose of STZ + 1 injection of insulin 30 min before sacrifice

**STZ HD Hypo group:** mice injected with 1 high dose of STZ that developed hypothermia.

### Physiological parameters of STZ injected hTau mice

We first measured key physiological parameters, including weight, glycaemia, insulinemia and body temperature (Table 1). One high dose of STZ induced significant weight loss of mice (groups: STZ HD, STZ HD Insulin, and STZ HD Hypothermic) while five low dose injections did not. As expected, STZ injected mice (high dose and low dose injections groups) exhibited hyperglycaemia along with obvious hypoinsulinemia in comparison with control mice. Physiological glycaemia was restored in mice injected with insulin 30 minutes before sacrifice. Interestingly, 7 mice injected with a high dose of STZ exhibited a significant drop in body temperature (34.8 ± 0.6 *vs.* 36.9 ± 0.5 °C) and were analyzed as a distinct group of STZ HD-injected mice (STZ HD Hypo) as hypothermia can impact on tau phosphorylation^26^.

### Tau hyperphosphorylation in STZ injected hTau mice

We tested whether type 1 diabetes induced tau phosphorylation in the hippocampus, using several anti-phospho-tau antibodies (Table S1). The specificity of the tau antibodies used in this study has been extensively characterized in our laboratory^27^. We first evaluated whether STZ low doses injections, inducing mild T1DM, led to tau hyperphosphorylation (STZ LD group). We observed significant tau hyperphosphorylation at AT8 (pSer202/pThr205), PHF-1 (pSer396/Ser404) and pThr205 epitopes (Fig. 2, panels 2, 3, 5). We also noticed a significant decrease of Tau-1 signal, which recognize non-phosphorylated tau at Ser195/Ser198/Ser199/Ser202 (Fig. 2, panel 6). A decrease of Tau-1 signal reflects tau hyperphosphorylation. We next examined tau phosphorylation in a group with marked T1DM, due to a single high dose injection of STZ (STZ HD group). This group also exhibited tau hyperphosphorylation according to Tau-1, AT8 and PHF1 signals but not to pT205 (Fig. 2, panels 5 & 6). Seven mice of the STZ HD group developed hypothermia following STZ injection (STZ HD Hypo group). As mentioned before, we separated these mice in a distinct group since hypothermia induces tau hyperphosphorylation^26^. Tau hyperphosphorylation on all tau phospho-epitopes tested (CP13, AT8, pT205, AT180, PHF-1, and, Tau-1) was observed in this group in comparison with controls (Fig. 2, panels 1 to 6) by western blot. We confirmed tau hyperphosphorylation in high dose groups by immunohistochemistry (Fig. 3). Total tau and β-actin levels remained unchanged (Fig. 2, panels 7 & 8).

### Impact of insulin injection on tau phosphorylation in STZ injected hTau mice

Since STZ injection lead to hypoinsulinaemia resulting in hyperglycaemia, we next tested whether insulin injection, restoring physiological insulinaemia and glycaemia, impacts on tau hyperphosphorylation in STZ injected mice (STZ LD Ins and STZ HD Ins groups). Interestingly, 30 minutes after insulin injection, physiological tau phosphorylation comparable to control mice was partially restored in both STZ LD Ins and STZ HD Ins groups on most phospho-epitopes exhibiting hyperphosphorylation (Fig. 2, panels 1 to 6). However, statistical significance between STZ-injected mice treated with or without insulin injection was observed only at Tau-1 phosphoepitope (STZ HD group *vs.* STZ HD Ins group) (Fig. 2.6). These results suggest that tau hyperphosphorylation in STZ-injected mice follows, at least in part, insulin deficiency in these mice. Total tau and β-actin levels remained unchanged (Fig. 2 panels 7 & 8). We confirmed the effect of insulin on tau hyperphosphorylation in STZ high dose groups by immunohistochemistry (Fig. 3). Insulin injection in control mice did not change tau phosphorylation (Fig. S1).

### Tau solubility in STZ injected hTau mice

As tau hyperphosphorylation has been shown to induce insoluble tau aggregation and tangle formation *in vitro*^17^, we analyzed insoluble and soluble fraction of tau in our different groups (Fig. 4). However, neither STZ nor insulin injections altered tau solubility (Fig. 4A,B).

### Tau kinases in STZ injected hTau mice

We next evaluated the mechanisms underlying tau hyperphosphorylation in STZ-injected mice. Tau hyperphosphorylation can be due to either activation of tau kinases, or inhibition of tau phosphatases. To test these two possibilities, we first examined a panel of major tau kinases activation/inhibition and expression (Table S1), including Ca2 + calmodulin-dependent protein kinase II (CamKII), c-Jun N-terminal kinase (JNK), P38 mitogen-activated protein kinase (P38), glycogen synthase kinase-3β (GSK-3β), mitogen activated protein kinase/extracellular signal-regulated kinase (ERK) and cyclin-dependent kinase 5 (cdk5) (Fig. 5). No change in tau kinases was observed in STZ-injected mice independently of insulin injection or hypothermia (Fig. 5, panels 2, 6, 8, 10, 11, 12). However, there was an increase of GSK-3β phosphorylation at Ser9 (indicating inhibition) in STZ HD and STZ HD hypothermic groups when compared to control mice (Fig. 5 panel 5), confirming previous reports^22^,^23^. Physiological Ser9 phosphorylation of GSK-3β comparable to control mice was restored after insulin injection (Fig. 5, panel 5). GSK-3β inhibition was, however, inconsistent with the elevation of tau phosphorylation in STZ-injected mice.

### Tau phosphatases in STZ injected hTau mice

As our prior results failed to explain tau hyperphosphorylation, we next turned to tau Ser/Thr protein phosphatases (PP) in the hippocampus, including PP1, PP2A, PP2B (calcineurin), and PP5^28^. All of them are highly expressed in the mammalian brain^29^. Biochemical studies have previously demonstrated that all these PP can dephosphorylate tau *in vitro*^30^. Therefore, we examined the profiles of these four PP using specific antibodies (Table S1). We observed no change in PP1, PP2B and PP5 expression in STZ injected hTau mice (Fig. 6, panels 1, 7, 8). We explored further the PP2A components since STZ injection has previously been show to reduce PP2A activity in the brain^22^,^23^. We thus investigated the levels of PP2A subunits: PP2A-A, PP2A-B (α and β, PP2A-C expression levels, as well as the demethylated PP2A-C (inactivated PP2A) in STZ injected hTau mice. No change was observed on PP2A subunits expression as well as its demethylation (Fig. 6, panels 2 to 6). We next analyzed PP2A activity with a commercial assay kit. After immunoprecipitation of PP2A from the samples, this assay measures the phosphates released from a synthetic phosphopeptide, which corresponds to the activity of PP2A in each sample. This assay revealed a decrease of PP2A activity in all STZ-injected groups (Fig. 6, panel 11) because fewer phosphates were released in these groups, suggesting that tau hyperphosphorylation reported in STZ-injected mice could be a consequence of PP2A inhibition. On the other hand, insulin injection did not restore physiological PP2A activity in STZ-injected mice. These data suggest that the beneficial effect of insulin injection on tau hyperphosphorylation might go through a different pathway than restoring PP2A activity.

Finally, we assessed PTEN expression and phosphorylation since PTEN phosphatase as been previously shown to impact on insulin signaling and resistance^31^ and affect tau phosphorylation, aggregation, and binding to microtubules through PIP3 signalling pathway^32^. We observed no change on both PTEN expression and phosphorylation at serine 380 in STZ-injected mice compared to controls (Fig. 6, panels 9 & 10).

### Brain insulin signaling in STZ injected hTau mice

We also evaluated brain insulin signalling since impaired central insulin signaling can induce tau hyperphosphorylation^16^,^33^. We investigated the activation state of three keys protein of insulin signaling: insulin receptor (IR), Akt and PI3K kinases, as well as IGF1 receptor, since IGF1-signaling pathway can be activated by insulin. Interestingly, Akt is also a tau kinase^34^. No change was observed for these four proteins. However, AKT and insulin receptors had increased phosphorylation in STZ HD and STZ HD Hypothermic groups compared to controls, reflecting their activation (Fig. 7, panels 1 & 7). Increased phosphorylation was also found on PI3K in STZ HD hypothermic group (Fig. 7, panel 5). Surprisingly, this hippocampal insulin pathway activation in STZ HD groups disappeared after i.p. insulin injection (Fig. 7.1,7.7). These results suggest a deregulation of central insulin signaling pathway in peripheral hypoinsulinemic conditions.

#### Synaptic markers in STZ-injected hTau mice

We also tested some synaptic markers to assess synaptic integrity in STZ-injected mice, since synapse loss is an early event in AD^35^. As tau hyperphosphorylation is thought to trigger dendritic atrophy and/or synaptic alterations^36^, we evaluated pre- and post- synaptic connections by measuring synaptic proteins such as drebrin, synaptophysin, SNAP25 and PSD95 (Fig. 8). Interestingly, STZ injections impacted on both pre- and post-synaptic markers as there was a decrease in drebrin, and a tendency to decrease in SNAP25 (both STZ LD and STZ HD groups *vs.* CTL group) (Fig. 8, panels 1 & 3). Insulin injection restored drebrin reduction compared to control (Fig. 8, panel 1), while it increased SNAP25 when compared to STZ-injected groups (Fig. 8, panel 3). However, synaptophysin and PSD95 remained however unchanged regardless of mice treatment (Fig. 8, panel 2 and 4). These results suggest that T1DM could promote synaptic impairments, which might be restored by insulin injection.

## Discussion

In this study, we addressed whether insulin deprivation would induce tau hyperphosphorylation and aggregation by administration of different doses of STZ in hTau mice, a humanized tau transgenic mouse. We report that hypoinsulinemia induced tau hyperphosphorylation without affecting tau aggregation. Our data indicated that tau hyperphosphorylation following STZ injection is likely a consequence of PP2A inhibition. Interestingly, insulin injection restoring physiological glycaemia partially restored normal tau phosphorylation to control levels in STZ injected mice.

Previous studies have examined the impact of STZ injection resulting in hypoinsulinemia on tau phosphorylation in wild-type rodents to explore the link between AD and insulin dysfunction^9^,^22^,^23^,^24^,^25^. Most used a single high dose of STZ injection to quickly induce a marked T1DM in wild-type rodents, and reported tau hyperphosphorylation at several phospho-epitopes such as AT8, PHF1 and Tau-1^9^,^22^,^23^,^25^,^37^. Similarly, we observed tau hyperphosphorylation in STZ HD group at these epitopes. AT8 (Ser^202^/Thr^205^) is an antibody routinely used for the staging of AD-associated neurofibrillary pathology^38^, and the phosphorylation sites detected by PHF-1 (Ser^396^/Ser^404^) are associated with neurofibrillary tangle pathology^39^. Unfortunately, few of these studies monitored mice body temperature after STZ injection, which is important since STZ injection can induce hypothermia resulting in massive tau hyperphosphorylation^22^. Therefore, it is difficult to conclude if the tau hyperphosphorylation observed in studies without temperature monitoring is the result of T1DM *per se* or consequent hypothermia or both. To avoid this artefact, mice developing hypothermia after STZ injection formed a distinct group from the other mice. As expected, these mice displayed extensive tau hyperphosphorylation on all six epitopes tested here, while tau was hyperphosphorylated at only three epitopes in normothermic mice, underlying the importance of recording mice body temperature in diabetic models.

It is important to examine tau aggregation after diabetes induction, since hyperphosphorylation has been shown to induce the formation of insoluble tau aggregates and neurofibrillary tangle *in vitro*^17^. Transgenic mice overexpressing human tau are the tool of choice since murine tau does not readily aggregate; surprisingly, only one study has used such mice models. Ke *et al*. observed increased tau aggregation in pR5 mice overexpressing P301L mutant human tau after T1DM induction with 1 high dose of STZ (200 mg/kg)^37^. Interestingly, in hTau mice (overexpressing non-mutant human tau), we did not detect any change in tau solubility after STZ injection despite marked hyperphosphorylation. The differences between the two lines could be due to the fact that P301L tau mutant is more prone to aggregation than non-mutant tau. Indeed, the P301L mutation has been shown to accelerate the formation of paired helical filaments and promote β-sheet formation^40^,^41^,^42^. While hTau mice do develop tau aggregates, they do it slower than pR5 mice. Therefore, the lack of mutation on tau in our model could account for the lack of exacerbation observed here. However, this does not mean absence of toxic tau species. Indeed, some forms of hyperphosphorylated tau have been shown to have cytotoxic proprieties^43^, and the synaptic deficits present in many models of tauopathies are often observed before tau aggregation and tangle formation^44^,^45^,^46^. Here, tau hyperphosphorylation might have contributed to synaptic deficits, along with the effects of diabetes *per se*^47^,^48^.

Other studies used multiple lower doses of STZ to induce a milder T1DM^9^,^24^,^49^,^50^. The results from these multiple doses of STZ are more variable. For example, Kim *et al*. did not report change in tau phosphorylation after 5 low doses (5 × 50 mg/kg) of STZ in WT mice brains^9^, while Jolivalt *et al*. observed tau hyperphosphorylation in brain of mice after 2 injections of low dose (2 × 90 mg/kg) of STZ^24^. In our study, we observed a roughly equivalent hyperphosphorylation of tau in mice injected with one high dose or five low doses of STZ, suggesting that the important factor is the diabetic state.

At the mechanistic level, the results diverge in the literature to explain tau hyperphosphorylation after STZ injection. Some studies have linked tau hyperphosphorylation to an increased activity of GSK-3β, a major tau kinase^24^,^25^, while others have suggested inhibition of PP2A as the main mechanism^22^,^23^,^25^. In STZ-injected hTau mice, we confirmed PP2A inhibition in both STZ LD and STZ HD groups, suggesting that it underlies tau hyperphosphorylation in these groups. Moreover, tau hyperphosphorylation along with inhibition of PP2A was observed in NOD mice, a spontaneous mouse model of T1DM^51^. At the kinase level, the only change observed was an increase of GSK-3β inhibitory phosphorylation at Ser9 HD, a result opposite to what was observed by some studies^24^,^25^, but in line with other observations (for review ref. 16). This result might be explained by the decrease in PP2A activity, since the inhibition of PP2A increases GSK-3β Ser9 phosphorylation and inhibits its activity^52^,^53^. These results are in line with others demonstrating that when PP2A is inhibited, it is the dominant factor inducing tau hyperphosphorylation, overriding the inhibition of key tau kinases^54^,^55^. While it is difficult to give a definitive mechanistic explanation to tau hyperphosphorylation in STZ-injected mice because of discrepancies in previous studies, our data support the PP2A inhibition hypothesis.

Surprisingly, only two studies assessed central insulin signaling in STZ-injected rodents^24^,^25^, despite the fact that impaired central insulin signalling can induce tau hyperphosphorylation^16^,^33^. Both reported a decrease of AKT phosphorylation, a key protein of insulin signaling in STZ injected rodent^24^,^25^. Jolivalt *et al*. also observed a reduction of insulin receptor phosphorylation at Ser972. In our study, we observed opposite results: increased insulin receptor and AKT phosphorylation in STZ-injected mice. These results are in line with the inhibitory phosphorylation of GSK-3β at Ser9 observed in STZ-injected hTau mice. Interestingly, a similar AKT activation was observed in NOD mice, a spontaneous mouse model of T1DM^51^. These results suggest a deregulation of brain insulin signalling in T1DM mice models.

Finally, we assessed whether insulin injection can restore physiological tau phosphorylation in STZ-injected mice. Previous studies reported that insulin treatment is sufficient to abolish tau hyperphosphorylation in STZ-injected rodents^22^,^23^,^25^. Tau hyperphosphorylation was attenuated in STZ-injected mice after peripheral insulin injection 30 minutes before sacrifice, confirming a link between insulin homeostasis and tau phosphorylation. However, insulin injection significantly decreased tau hyperhosphorylation only at one phosphoepitope compared to STZ-injected mice without insulin injection 30 minutes before sacrifice. We could hypothesized that one acute insulin supplementation is not enough to totally restore physiological tau phosphorylation in mice deprived of insulin for 4 weeks. Indeed, previous studies observed a rescue of tau phosphorylation after daily insulin administration in STZ-injected rodents^22^,^25^. Interestingly, insulin did not restore physiological PP2A activity, suggesting the beneficial effect of insulin on tau hyperphosphorylation goes through different pathway. Interestingly, GSK-3β Ser9 levels in STZ-injected mice were restored to normal levels after insulin injection, alleviating its inhibition. Although it could not directly explain decrease of tau hyperphosphorylation after insulin injection, restoring GSK-3β indirectly contribute to the reduction of tau hyperphosphorylation by activating PP1. Indeed, GSK-3β is able to promote PP1 activity by phosphorylation of PP1 Inhibitor-2^56^,^57^. Further studies are needed to confirm this hypothesis. Insulin administration also increased synaptic markers reduced by STZ injection. This effect of insulin on synaptic markers as previously reported^58^,^59^,^60^ and insulin is known to regulate synapse number and dendritic plasticity^60^,^61^. Our data support that brain insulin dysregulation impacts on synaptic alterations and that tau hyperphosphorylation could contribute to this phenomenon.

To conclude, our study provides important data on the impact of insulin deprivation on tau phosphorylation and implicates the deregulation of PP2A activity (Fig. 9). Although the link between T1DM and Alzheimer’s disease is not as clearly established in humans as it is for T2DM^10^,^11^,^12^, these data provide valuable information by evaluating the effects of hyperglycemia and hypoinsulinemia seen in advanced stages of T2DM on AD pathology. Indeed, the patients in advanced stage of T2DM have higher risk of AD^11^. Therefore, Insulin deregulation resulting in decrease of PP2A activity and tau hyperphosphorylation might explain the higher tau phosphorylation and pathology found in T2DM patients and could underlie, at least in part, the high risk od AD in DM patients. These observations highlight the importance of glycaemia and insulinemia control in diabetic patients.

## Materials and Methods

### Animals

The founders of our hTau mice colony were purchased from the Jackson Laboratory (Bar Harbor, ME, USA) (B6.Cg-Mapttm1 (EGFP) Klt Tg(MAPT)8cPdav/J) on C57BL/6J background. They were initially generated by the group of Dr Peter Davies^62^ by crossing tau knockout mice that have a targeted disruption of exon one of murine tau^63^, with 8c mice that express a tau transgene containing the coding intronic and regulatory regions of the human gene^64^. These mice develop tau pathology in a time course and distribution comparable to that occurring in the early stages on human AD^62^.

7-9 months-old mice of either sex were maintained in a temperature-controlled room (~23 °C) with a light/dark cycle of 12/12 h. Experiments were performed during the light period. All animals had access to food and water *ad libitum*. Animals were handled according to procedures approved by the Comité de Protection des Animaux du CHUL under the guidelines of the Canadian Council on Animal Care.

### Streptozotocin and insulin injections

#### STZ

**Figure 1:**
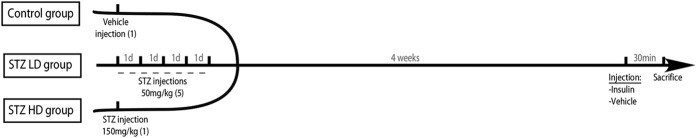
Experimental design of the study.

**Figure 2:**
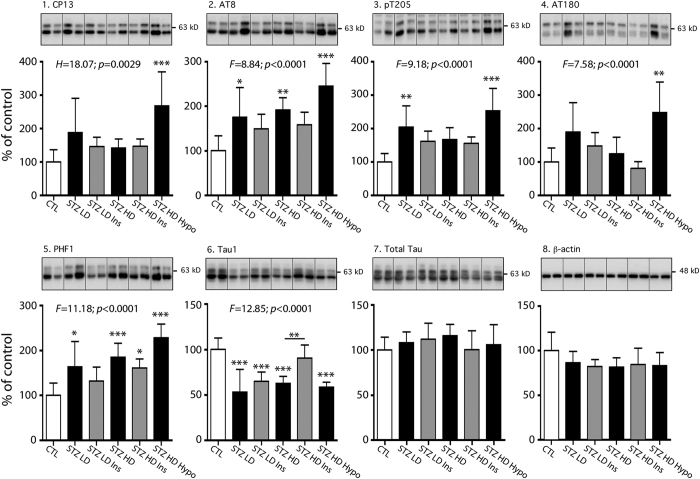
Tau phosphorylation in STZ-injected mice. Hippocampal proteins from 7–9 months-old mice were probed with the following antibodies: 1. CP13, 2. AT8, 3. pT205, 4. AT180, 5. PHF1, 6. Tau-1, 7. Total tau and 8. β-actin (loading control). Two lanes from representative immunoblots are displayed for each condition. Dividing lines represent areas where lanes from the same blot were removed and the remaining lanes were spliced together. Quantifications of tau phosphoepitopes were normalized to total tau levels. Total tau quantification was normalized to β-actin. Results are expressed as percentage of control group (CTL). Data are mean ± SD. Asterisks indicate significant differences, with *p < 0.05, **p < 0.01 and ***p < 0.001. Data from AT8, pT205, AT180, PHF1, Tau-1 and total tau following a normal distribution were analyzed with one-way ANOVA of variance followed by a Bonferroni’s *post hoc* test, while Kruskal-Wallis test followed by a Dunn’s *post hoc* test was used for CP13 and β-actin analysis since data deviated from a normal distribution.

**Figure 3:**
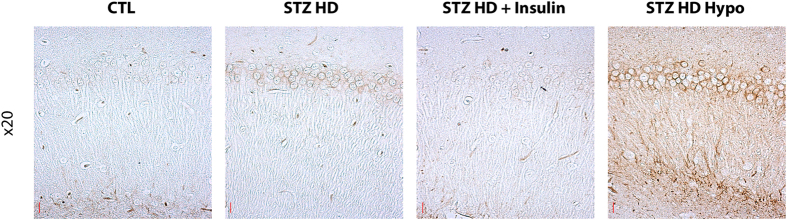
Immunostaining for phosphorylated tau in CA1 section of hippocampus. Regional anatomical localization of phosphorylated tau. Immunolabelling of hippocampal sagittal sections (CA1 region) are shown with AT8 for the following conditions: Control group, STZ HD group, STZ HD + Insulin group and hypothermic STZ-injected mice. Red scale bar: 20μm.

**Figure 4:**
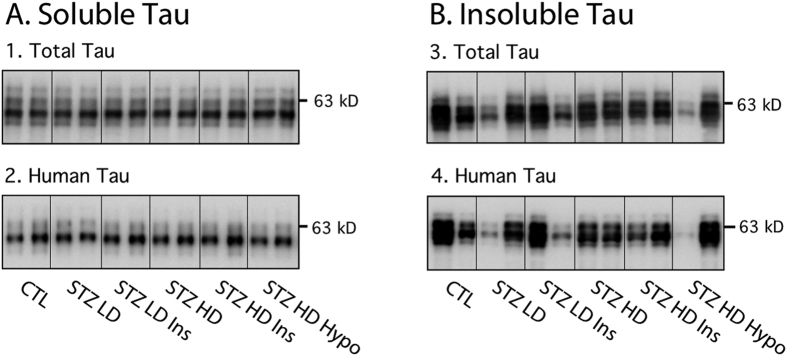
Tau solubility in STZ-injected mice. A. Soluble tau: Cortical soluble tau were probed with the following antibodies: 1. Total tau and 2. Human tau. B. Insoluble tau: Cortical aggregated of tau protein were probed with the following antibodies: 1. Total tau and 2. Human tau. Two lanes from representative immunoblots are displayed for each condition. There was no significant difference between STZ-injected mice and their controls (quantification data not shown). Data from insoluble total and human tau and soluble human tau following a normal distribution were analyzed with one-way ANOVA of variance followed by a Bonferroni’s *post hoc* test, while Kruskal-Wallis test followed by a Dunn’s *post hoc* test was used for soluble total tau analysis since data deviated from a normal distribution.

**Figure 5:**
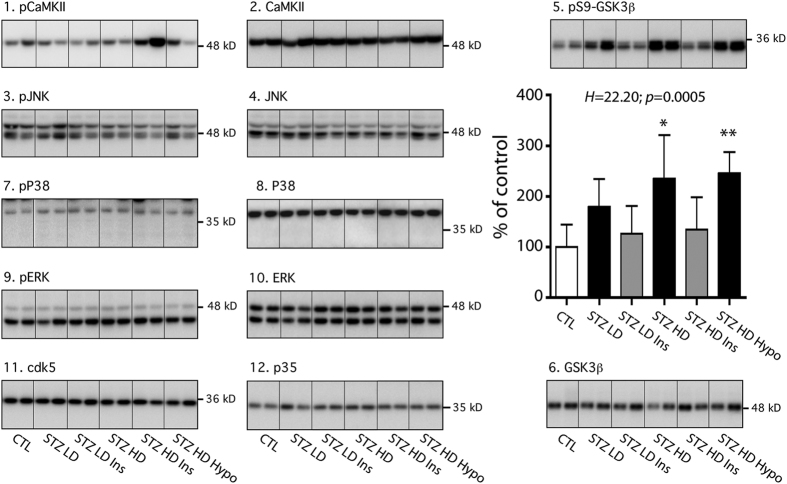
Tau kinases in STZ-injected mice. Hippocampal proteins from 7–9 months-old mice were probed with the following antibodies: 1. pCamKII, 2. CamKII, 3. pJNK, 4. JNK, 5. pS9-GSK3β, 6. GSK3β, pP38, 8. P38, 9. pERK, 10. ERK, 11. cdk5, 12. P35. Two lanes from representative immunoblots are displayed for each condition. Dividing lines represent areas where lanes from the same blot were removed and the remaining lanes were spliced together. Quantifications of phosphoepitopes were normalized to total form of respective protein. Total protein expression was quantified *vs.* β-actin. Except for pS9-GSK3β, there was no difference between STZ-injected mice and their controls (quantification data not shown). Results of pS9-GSK3β quantification are expressed as percentage of control group (CTL). Data are mean ± SD. Asterisks indicate significant differences from controls, with *p < 0.05 and **p < 0.01. Data from CamKII, pJNK, JNK, pERK, ERK, cdk5 and p35 following a normal distribution were analyzed with one-way ANOVA of variance followed by a Bonferroni’s *post hoc* test, while Kruskal-Wallis test followed by a Dunn’s *post hoc* test was used for pCaMKII, pP38, P38, pS9-GSK3β GSK3β analysis since data deviated from a normal distribution.

**Figure 6:**
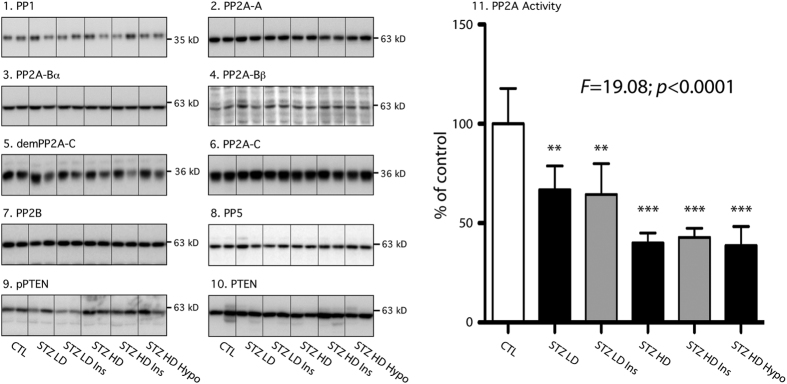
Tau phosphatases in STZ-injected mice. Hippocampal proteins from 7–9 months-old mice were probed with the following antibodies: 1. PP1, 2. PP2A-A, 3. PP2A-Bα, 4. PP2A-Bβ, 5. demPP2A-C, 6. PP2A-C, 7. PP2B, 8. PP5, 9. pPTEN, 10. PTEN. Two lanes from representative immunoblots are displayed for each condition. Dividing lines represent areas where lanes from the same blot were removed and the remaining lanes were spliced together. Quantification of demethylated or phosphorylated phosphatase was normalized to total phosphatase. Total protein expression was quantified *vs.* β-actin. There was no significant difference between STZ-injected mice and their controls (quantification data not shown). 11. PP2A activity: Results are expressed as percentage of control group (CTL). Data are mean ± SD. Asterisks indicate significant differences from controls, with **p < 0.01 and ***p < 0.001. Data from PP1, PP2A-Bβ, demPP2A-C, PP2A-C, pPTEN and PP2A activity following a normal distribution were analyzed with one-way ANOVA of variance followed by a Bonferroni’s *post hoc* test, while Kruskal-Wallis test followed by a Dunn’s *post hoc* test was used for PP2A-A, PP2A-Bα, PP2B, PP5 and PTEN analysis since data deviated from a normal distribution.

**Figure 7:**
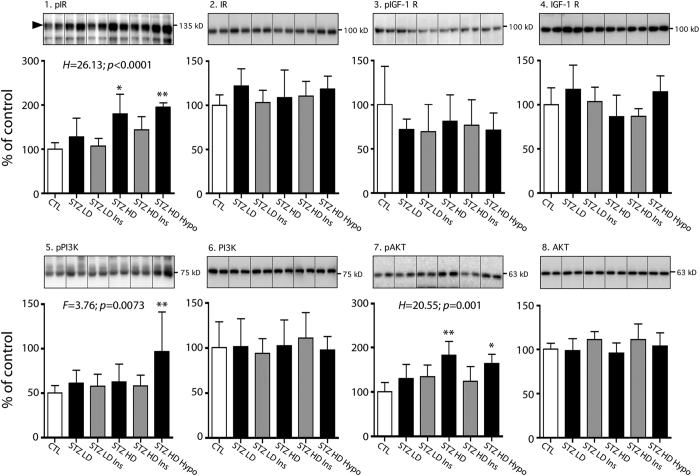
Central insulin signaling in STZ-injected mice. Hippocampal proteins from 7–9 months-old mice were probed with the following antibodies: 1. pIR, 2. IR, 3. pIGF1 Rc, 4. IGF1 Rc, 5. pPI3K, 6. PI3K, 7. pAKT, 8. AKT. Two lanes from representative immunoblots are displayed for each condition. Dividing lines represent areas where lanes from the same blot were removed and the remaining lanes were spliced together. Quantifications of phosphoepitopes were normalized to total form of respective protein. Total protein expression was quantified *vs.* β-actin. Results are expressed as percentage of control group (CTL). Data are mean ± SD. Asterisks indicate significant differences from controls, with *p < 0.05 and **p < 0.01. Data from pIGF-1 Rc, IGF-1 Rc, pPI3K, PI3K and AKT following a normal distribution were analyzed with one-way ANOVA of variance followed by a Bonferroni’s *post hoc* test, while Kruskal-Wallis test followed by a Dunn’s *post hoc* test was used for pIR, IR and pAKT analysis since data deviated from a normal distribution.

**Figure 8:**
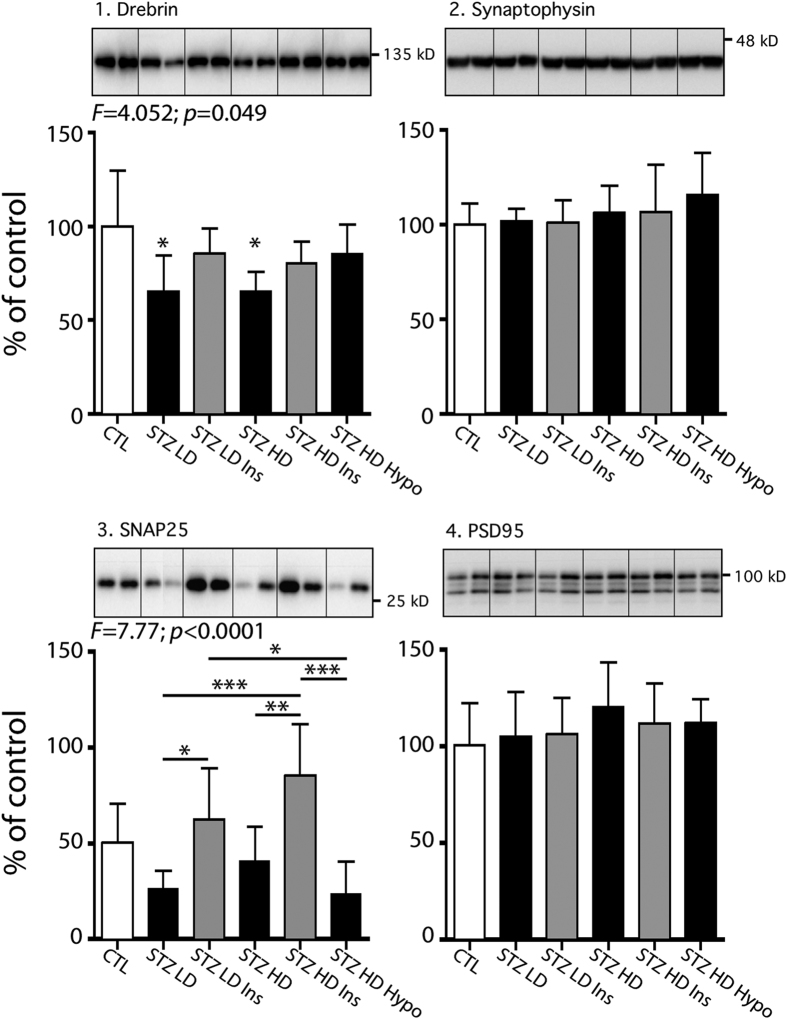
Synaptic markers in STZ-injected mice. Hippocampal proteins from 7–9 months-old mice were probed with the following antibodies: 1. Drebrin, 2. Synaptophysin, 3. SNAP25, 4. PSD95. Two lanes from representative immunoblots are displayed for each condition. Dividing lines represent areas where lanes from the same blot were removed and the remaining lanes were spliced together. Total protein expression was quantified *vs.* β-actin. Results are expressed as percentage of control group (CTL). Data are mean ± SD. Asterisks indicate significant differences, with *p < 0.05 and **p < 0.01. Data from this figure following a normal distribution were analyzed with one-way ANOVA of variance followed by a Bonferroni’s *post hoc* test.

**Figure 9:**
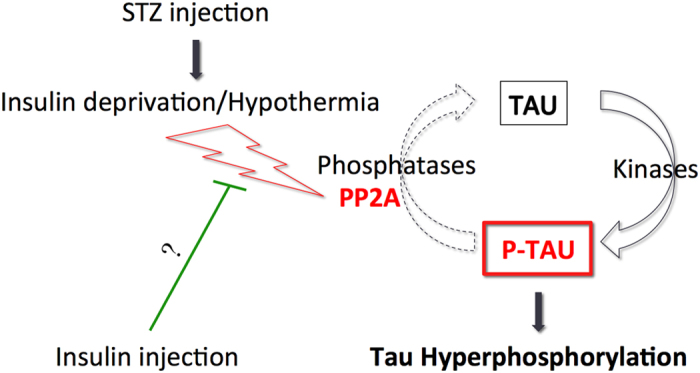
Putative mechanism of tau hyperphosphorylation during insulin deprivation. STZ injection induces insulin deprivation by destroying insulin-producing cells in pancreas. The resulting T1DM lead to PP2A inhibition in the brain, which induce tau hyperphosphorylation. Insulin injection 30 minutes before sacrifice partially restores physiological phosphorylation of tau comparable to non-T1DM mice without rescue of PP2A activity, suggesting involvement of different pathway.

Nonfasted animals were injected following two different protocols from the Animal Models for Diabetic Complications Consortium (www.diacomp.org). Two groups of mice received one intraperitoneal injection of 50 mg/kg of STZ (2-deoxy-2-(3-(methyl-3-nitrosoureido)-D-glucopyranose); Sigma, St. Louis, MO) per day for 5 consecutive days according with Multiple Low-Dose STZ induction Protocol (STZ LD groups). This protocol induces a mild and progressive T1DM that can last for many months without overt toxicity for the mice. Two other groups received only one intraperitoneal injection of 150 mg/kg of STZ (Sigma, St. Louis, MO) according with the High-Dose STZ induction Protocol (STZ HD groups). This protocol quickly induces a marked T1DM. STZ was freshly dissolved in 0.05 M citrate buffer, pH 4.5. Vehicle-injected mice were used as (Fig. 1) controls.

#### Insulin

Thirty minutes before mice sacrifice, half of each STZ group (STZ-HD and STZ-LD) received insulin injection (4 IU/kg) to reverse the hypoinsulinemic effects of STZ. The second half of each group and control group received injection of 0.9% saline as vehicle. For control purpose, the same insulin and vehicle injections was done in non-diabetic hTau mice (n = 8 for each group) to evaluate the specific effect of insulin on tau phosphorylation.

#### Physiological parameters

All mice were weighed at the time of sacrifice, and body temperature was monitored using a rectal probe (Thermalert TH-5, Physitemp, Clifton, NJ). Fasting blood glucose and insulin were measured using a glucometer with reagent strips (ACCU-CHEK ^®^ Aviva Nano; Roche Diagnostics GmbH, Mannheim, Germany) and a sandwich enzyme immunoassay (Ultrasensitive Mouse Insulin ELISA, Mercodia, Sweden) respectively. Weight, temperature fasting blood glucose and insulin of mice group are reported in Table 1.

#### Protein extraction

Four weeks after STZ injections, the mice were killed by decapitation without anaesthesia, as anaesthesia can lead to hypothermia-induced tau hyperphosphorylation^65^. Brains were immediately removed and the tissues dissected on ice, frozen on dry ice, and kept at −80 °C until they were processed as described^66^. Briefly, dissected brain structures (hippocampus and cortex) were homogenized, without thawing, in 5 times volume/weight of radioimmunoprecipitation assay (RIPA) buffer (50 mM Tris-HCl, pH 7.4, 1% NP-40, 150 mM NaCl, 0.25% Na-deoxycholate, 1 mM EDTA, 1 mM Na_3_VO_4_, 1 mM NaF, 1 mM PMSF, 10 μl/ml of Proteases Inhibitors Cocktail (P8340, Sigma-Aldrich, St. Louis, MO)), using a mechanical homogenizer (TH, Omni International, Marietta, GA). Samples were then centrifuged for 20 min at 20,000 g at 4 °C. The supernatant was recovered, diluted in sample buffer (NuPAGE LDS; Invitrogen, Carlsbad, CA) containing 5% of 2-β-mercapto-ethanol, 1 mM Na_3_VO_4_, 1 mM NaF, 1 mM PMSF, 10 μl/ml of Proteases Inhibitors Cocktail (P8340; Sigma-Aldrich), boiled for 5 min. and kept at −20 °C.

#### Purification of aggregates

Tau aggregates were extracted according to our protocol previously used to isolate tau aggregates in mouse models of tauopathies^67^. This procedure uses 1% sarkosyl and is derived from one used to isolate tau aggregates from the brains of AD^68^.

Briefly, the RIPA supernatant was adjusted to 1% sarkosyl (*N*-lauroylsarcosine), incubated for 30 min at room temperature with constant shaking, and centrifuged at 100,000 × g for 1 hour at 20 °C. The pellet containing sarkosyl-insoluble aggregated (SP fraction) was resuspended and diluted in Sample buffer (NuPAGE LDS) containing 5% of 2-β-mercapto-ethanol, 1 mM Na_3_VO_4_, 1 mM NaF, 1 mM PMSF, 10 μl/ml of Proteases Inhibitors Cocktail (P8340, Sigma-Aldrich), boiled for 5 min, and kept at −20 °C.

For heat stable soluble tau, the RIPA supernatant was boiled for 5 min and centrifuged at 20,000 × g for 20 min. The supernatant was recovered, diluted in sample buffer (NuPAGE LDS; Invitrogen, Carlsbad, CA) containing 5% of 2-β-mercapto-ethanol, 1 mM Na_3_VO_4_, 1 mM NaF, 1 mM PMSF, 10 μl/ml of Proteases Inhibitors Cocktail (P8340; Sigma-Aldrich) and boiled for 5 min.

#### Western-blot analysis

SDS-PAGE and Western blot analysis were done as previously described^27^. All antibodies used in this study are listed in Table 1. Depending on the antibody used, 5–15 μg of brain protein were analyzed. Brain homogenates were separated on a SDS-10% polyacrylamide gel and then transferred onto nitrocellulose membranes (Amersham Biosciences, Pittsburgh, PA). Non-specific binding sites were blocked with 5% nonfat dry milk in Phosphate-buffered saline containing 0.1% Tween 20 (PBS-T) for one hour at room temperature and were afterwards incubated overnight at 4 °C with primary antibodies directed against the specific antibody. The following day, membranes were washed 3 times in PBS-T and then incubated for 1 hour at room temperature with the corresponding secondary antibody in 5% nonfat dry milk in PBS-T, and the immunoreactive signal intensity was visualized by enhanced chemiluminescence (ECL Plus, GE Healthcare Biosciences, Piscataway, NJ). Some anti-tau monoclonal antibodies were revealed with anti-LightChain secondary antibody according to our previous study^27^. Immunoreactive bands were visualized using ImageQuant LAS 4000 imaging system (Fujifilm USA, Valhalla, NY) and densitometric analyses were performed with Image Gauge analysis software (Fujifilm USA, Valhalla, NY).

#### Immunohistochemistry

Tissue fixation was done according to the “Cold Bouin’s method”^69^. Briefly, mice were sacrificed by decapitation, the brain was quickly removed and immersed in ice-cold Bouin’s solution (saturated picric acid, formalin, acetic acid at 15:5:1) for 24 h and then embedded in paraffin blocks. Five μm thick sections were processed for immunostaining analyses. Prior to immunostaining, slides were deparaffinized with Citrisolv for 2 × 10 minutes. The sections were subsequently rehydrated by immersing them in graded ethanol solutions (100%, 95%, 70% and 50%) during 5 minutes for each condition. After further rinses in water, deparaffinized and hydrated sections were incubated for 1 hour in citrate buffer previously warmed at 80°C for enhancement of the immunoreactivity. The slides were next washed in PBS 0, 1 M (pH 7.4) for 3 × 10 minutes, then incubated in a solution of PBS containing 3% of peroxide for 30 minutes, and washed again in PBS for 3 × 10 minutes. The specimens were blocked for 30 minutes with a solution of PBS containing 5% NGS and 1% of Triton X-100 10%. The sections were next incubated overnight in primary antibodies diluted in 5% NGS in PBS 0.1 M (pH 7.4) containing 1% of Triton X-100 10% at 4°C. Bound antibodies were visualized with biotinylated anti-rabbit or anti-mouse IgG (Vector Laboratories Inc., Burlingame, CA). Immunolabeled tissues were observed under a Carl Zeiss AxioCamIC (Carl Zeiss, Jena, Germany) microscope.

#### PP2A activity assay

Brain PP2A activity was evaluated using a kit from R&D Systems according to the manufacturer’s instructions (Human/Mouse/Rat Active PP2A DuoSet IC, R&D Systems, Minneapolis, MN, USA). Briefly, after immunoprecipitation of PP2A for each sample, PP2A activity was determined by measuring the released phosphate from a chemically synthetized phosphopeptide after 30 minutes of incubation at 37°C. The level of phosphate release corresponds to PP2A activity and was determined by the absorbance of malachite green-phosphate at 620 nm.

#### Statistical analysis

Statistical analyses were performed with GraphPad Prism software 4.0 (Graphpad Software, La Jolla, CA). We used one-way ANOVA of variance followed by a Bonferroni’s *post hoc* test, if the effects were significant (*p* < 0.05) as assessed by ANOVA under normal distribution. If samples deviated from a normal distribution, the statistical analysis was performed using Kruskal-Wallis test followed by a Dunn’s *post hoc* test, when indicated (*p* < 0.05). For figure S1, statistical analyses were performed using unpaired *t*-tests. *, **, and *** symbols indicate significant differences *vs.* control with *p* < 0.05, *p* < 0.01, *p* < 0.001, respectively. Quantitative data were presented as mean ± SD.

## Additional Information

**How to cite this article:** Gratuze, M. *et al*. Insulin deprivation induces PP2A inhibition and Tau hyperphosphorylation in hTau mice, a model of Alzheimer’s disease-like tau pathology. *Sci. Rep.* **7**, 46359; doi: 10.1038/srep46359 (2017).

**Publisher's note:** Springer Nature remains neutral with regard to jurisdictional claims in published maps and institutional affiliations.

## Supplementary Material

Supplementary Dataset 1

Supplementary Information

## Figures and Tables

**Table 1:** Physiological parameters.

	Control (n = 6)	STZ LD (n = 7)	STZ LD Ins (n = 8)	STZ HD (n = 8)	STZ HD Ins (n = 8)	STZ HD Hypo (n = 7)
Weight (g)	34.1 ± 6.4	28 ± 5.4	31.2 ± 8.4	23.6 ± 2.6*	23.6 ± 2.5*	22.9 ± 4.4**
Glycemia (mmol/L)	11.6 ± 1.4	29.7 ± 3***	9.9 ± 8.6	25.1 ± 3.3***	12.1 ± 7.1	27 ± 4***
Insulinemia (μg/L)	1.41 ± 0.39	0.14 ± 0.07***	+++	0.03 ± 0.03***	+++	0.009 ± 0.004***
Temperature (°C)	36.9 ± 0.5	36 ± 0.9	36.3 ± 0.7	37.2 ± 0.5	37.2 ± 0.4	34.8 ± 0.6***

Body weight, fasting glycaemia and insulinemia and body temperature. Data are mean ± SD. Asterisks indicate significant differences from controls, with *p < 0.05, **p < 0.01 and ***p < 0.001. Data from physiological parameters following a normal distribution were analyzed with one-way ANOVA of variance followed by a Bonferroni’s *post hoc* test.
