# Supplementary material for: Insulin deprivation induces PP2A inhibition and tau hyperphosphorylation in hTau mice, a model of Alzheimer’s disease-like tau pathology
Source: Sci Rep. 2017 Apr 12;7:46359. doi: 10.1038/srep46359 (PMC5389355; doi:10.1038/srep46359)
Supplement: Supplementary Dataset 1 [file srep46359-s1.doc]

**SUPPLEMENTARY DATA**

**Insulin deprivation induces PP2A inhibition and tau hyperphosphorylation in hTau mice, a model of Alzheimer’s disease-like tau pathology**

Maud Gratuze ^a,b^, Jacinthe Julien ^b^, Franck R. Petry ^a,b^, Françoise Morin ^b^, Emmanuel Planel ^a,b^,*

^a^ Université Laval, Faculté de médecine, Département de Psychiatrie et Neurosciences, Québec, QC, Canada

^b^ Centre de Recherche du CHU de Québec, Axe Neurosciences, Québec, QC, Canada

* Corresponding author at: CHUL, RC-9800, 2705 Boulevard Laurier, Québec, QC, Canada, G1V 4G2. Tel. : +1 418 525 4444 #47805 ; fax : +1 418 654 2753.

*E-mail adress*: emmanuel@planel.org (E.Planel).


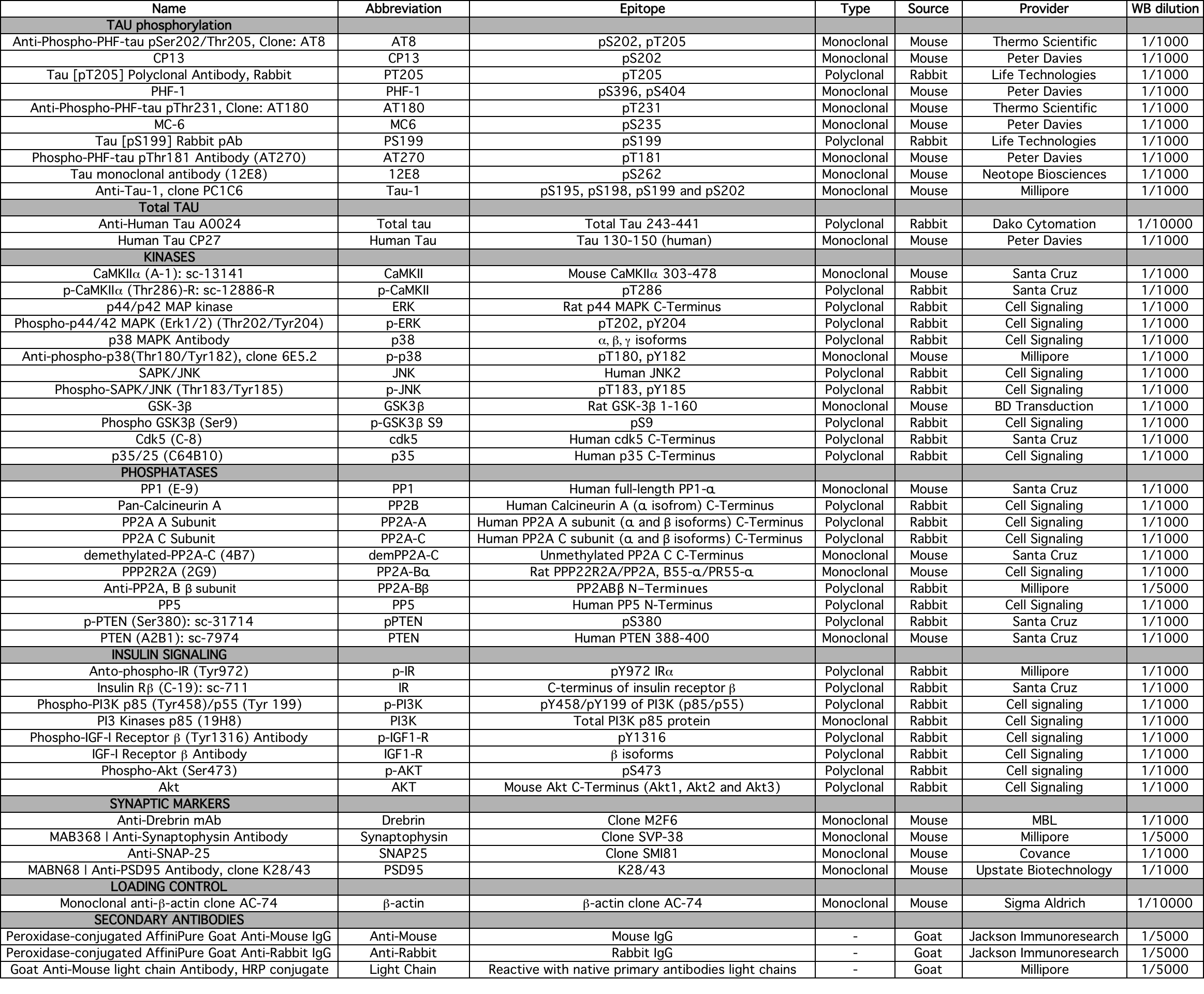


**Table S1:** Antibodies used in this study.

**
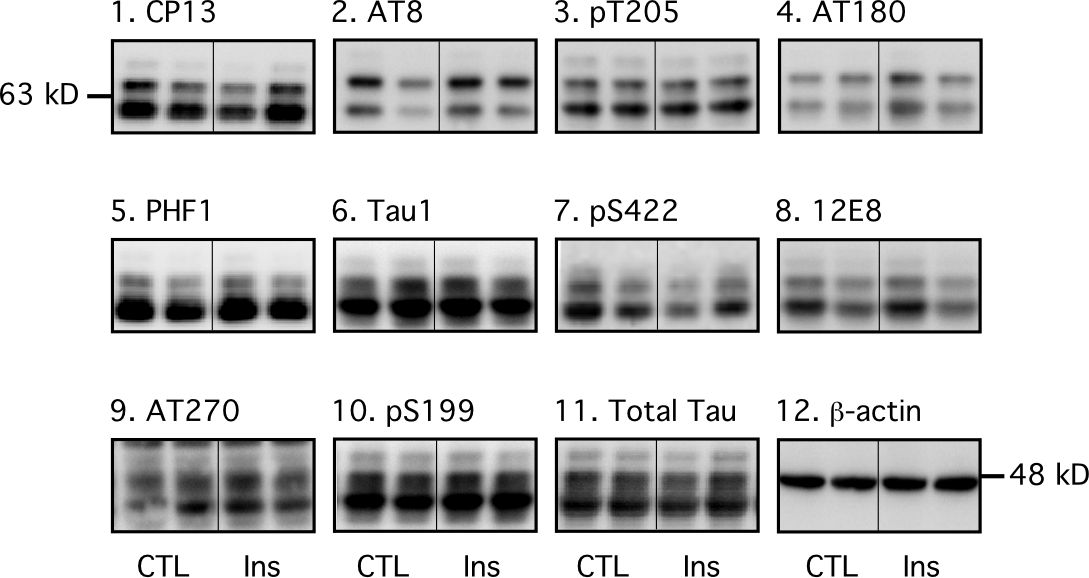
**

**Figure S1 :** Tau phosphorylation in insulin-injected mice. Hippocampal proteins from 7-10 months-old mice were extracted, separated by SDS-PAGE, and probed with the following antibodies: 1. CP13, 2. AT8, 3. pT205, 4. AT180, 5. PHF-1, 6. Tau1, 7. pS422, 8. 12E8, 9. AT270, 10. pS199, 11. Total Tau and 12. β-actin (loading control). Two lanes from representative immunoblots are displayed for each condition (from a n=8). Dividing lines represent areas where lanes from the same blot were removed and the remaining lanes were spliced together. There was no significant difference for all the phospho-epitopes between insulin-injected mice (4IU/kg i.p. 30 min before sacrifice) and their controls (quantification data not shown).
