# Supplementary material for: Insulin deprivation induces PP2A inhibition and tau hyperphosphorylation in hTau mice, a model of Alzheimer’s disease-like tau pathology
Source: Sci Rep. 2017 Apr 12;7:46359. doi: 10.1038/srep46359 (PMC5389355; doi:10.1038/srep46359)
Supplement: Supplementary Information [file srep46359-s2.pdf]

SUPPLEMENTARY INFORMATION

Insulin deprivation induces PP2A inhibition and tau hyperphosphorylation in hTau mice, a model of Alzheimer's disease-like tau pathology

Maud Gratuze [a,b], Jacinthe Julien [b], Franck R. Petry [a,b], Françoise Morin [b], Emmanuel Planel [a,b,*]

a Université Laval, Faculté de médecine, Département de Psychiatrie et Neurosciences, Québec, QC, Canada
b Centre de Recherche du CHU de Québec, Axe Neurosciences, Québec, QC, Canada

* Corresponding author at: CHUL, RC-9800, 2705 Boulevard Laurier, Québec, QC, Canada, G1V 4G2. Tel. : +1 418 525 4444 #47805 ; fax : +1 418 654 2753.
E-mail adress: emmanuel@planel.org (E.Planel).

	ID souris	
CTL (Saline)	004234	
	004222	
	004383	
	004421	
	004433	
	004585	
STZ low dose (Saline)	004201	
	004232	
	004303	
	004329	
	004381	
	004465	← Tau KO
	004430	
	004562	
STZ LD + Insuline 4IU/kg)	004202	
	004237	
	004252	
	004309	
	004432	
	004569	
	004539	
	004570	
STZ High dose	004177	
	004217	
	004376	
	004878	
	004920	
	004923	
	004871	
	004904	
STZ HD + Insuline	004178	
	004206	
	004331	
	004485	
	004530	
	004584	
	004879	
	004909	
STZ High dose hypothermique	004471	
	004544	
	004496	
	004491	
	004542	
	004889	
	004918	

Figure 2

1. CP13

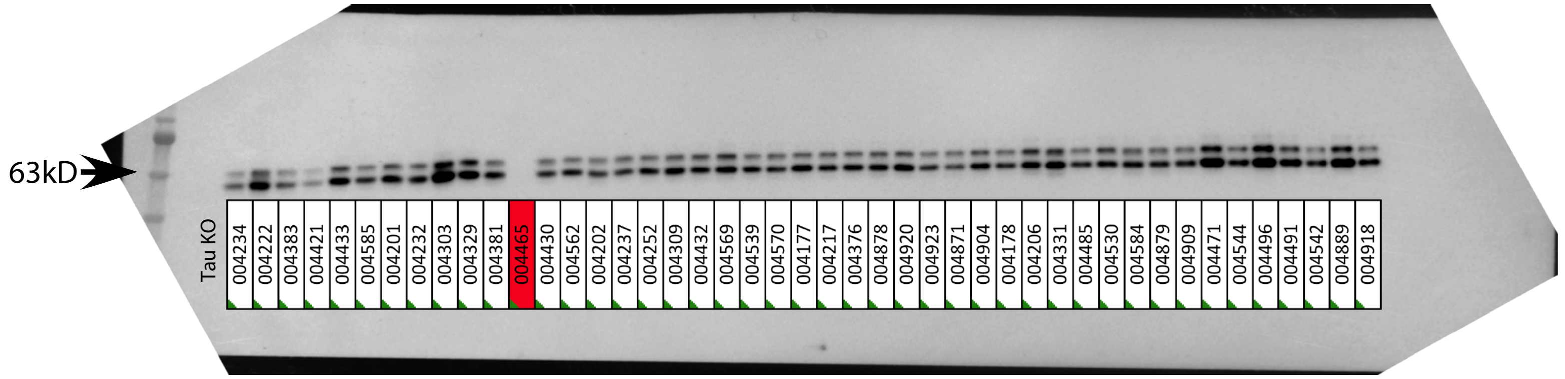

2. AT8

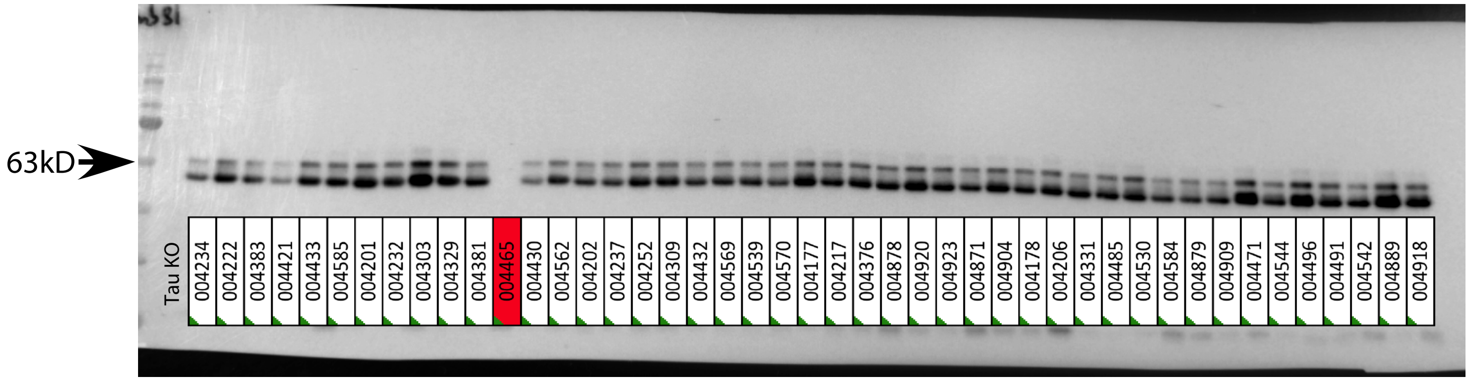

3. pT205

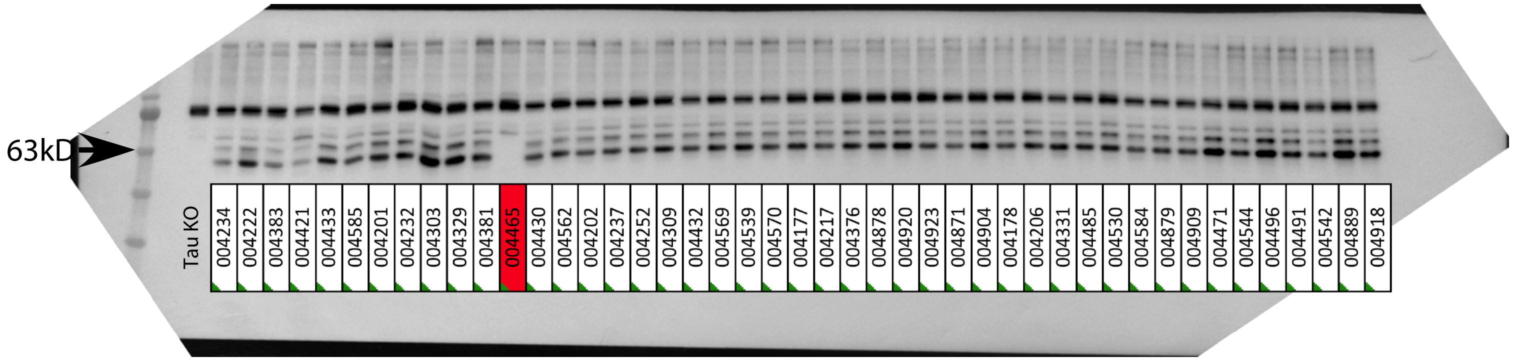

4. AT180

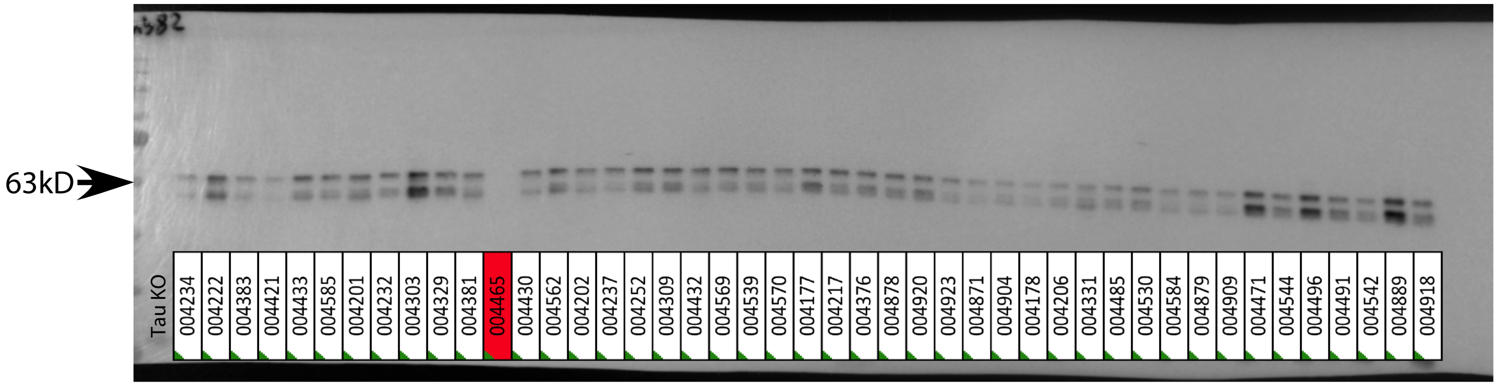

5. PHF1

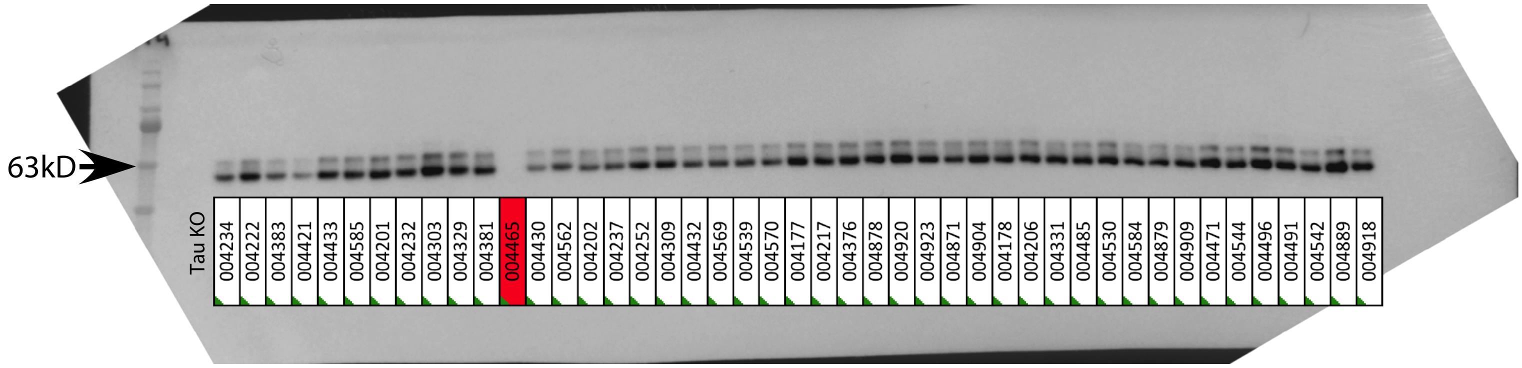

6. Tau1

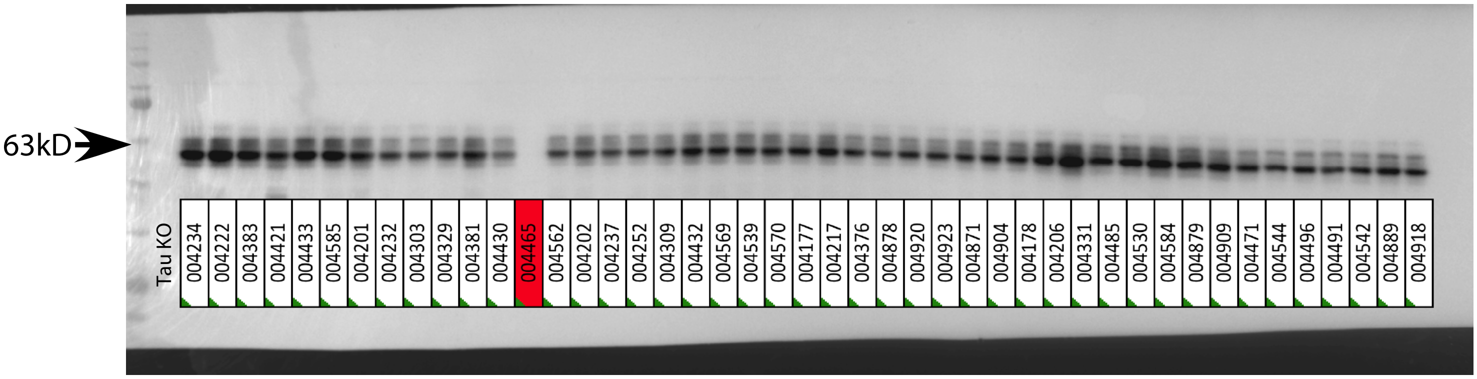

7. Total Tau

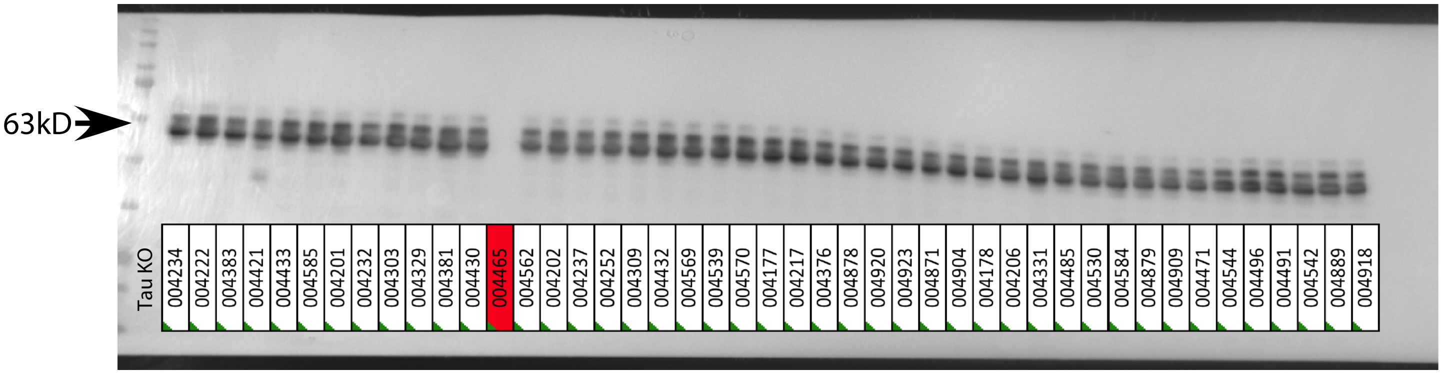

8. β-actin

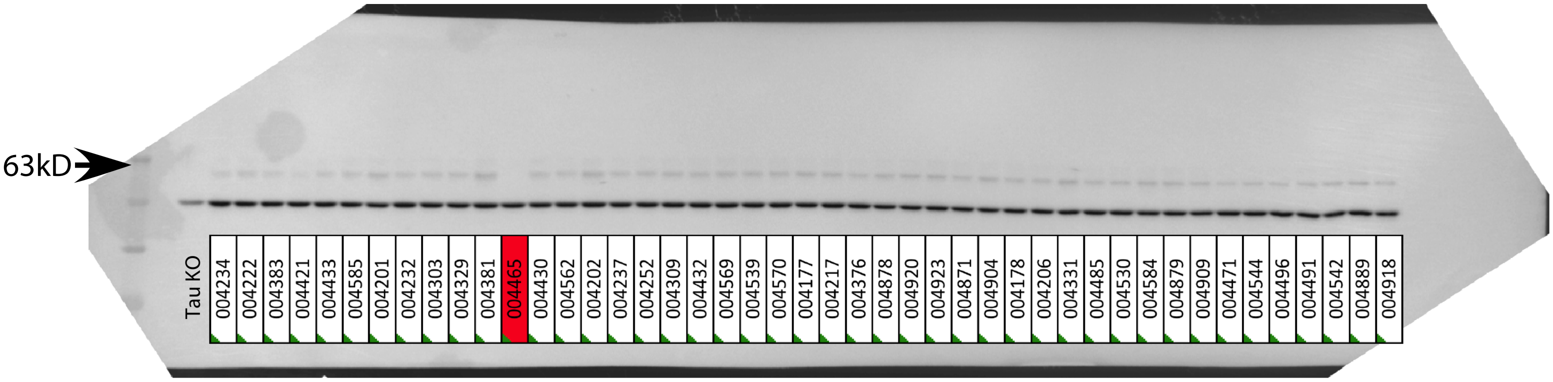

Figure 4

A. Soluble Tau

1. Total Tau

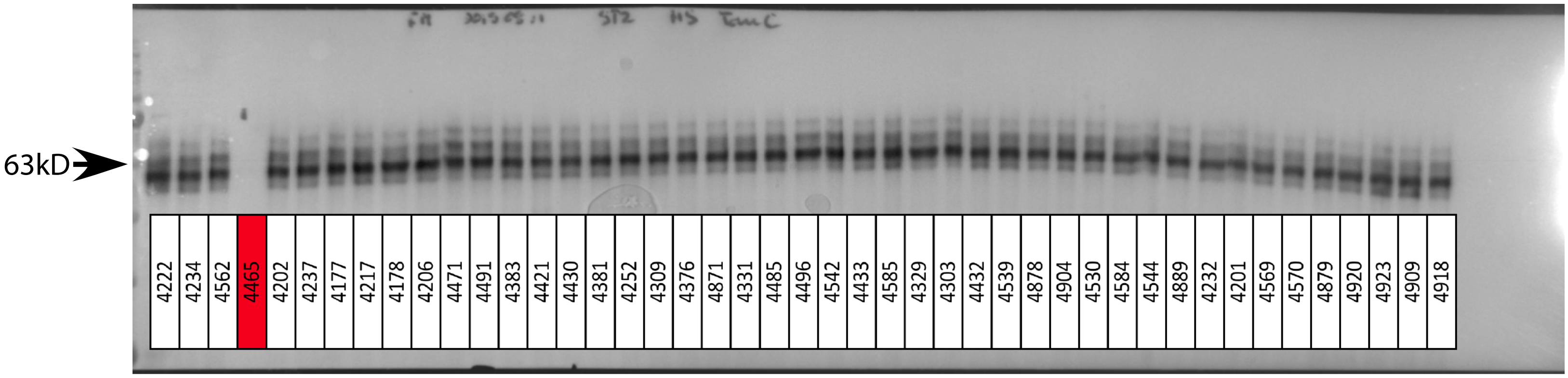

2. Human Tau

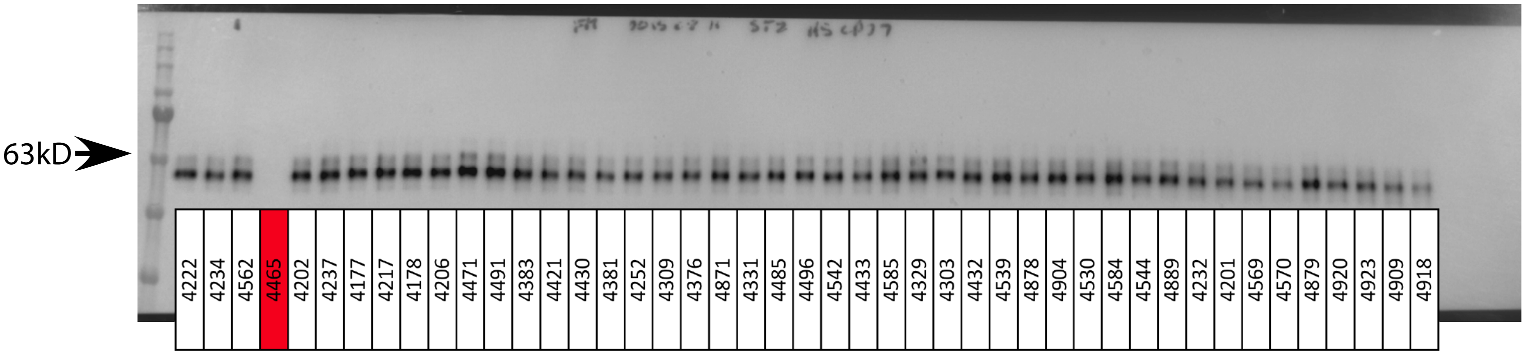

B. Insoluble Tau

3. Total Tau

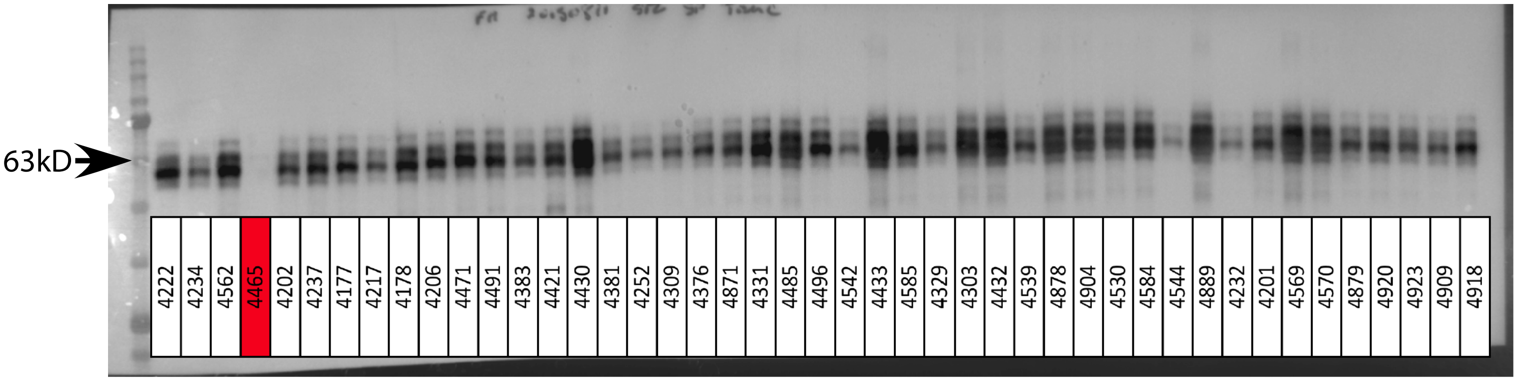

4. Human Tau

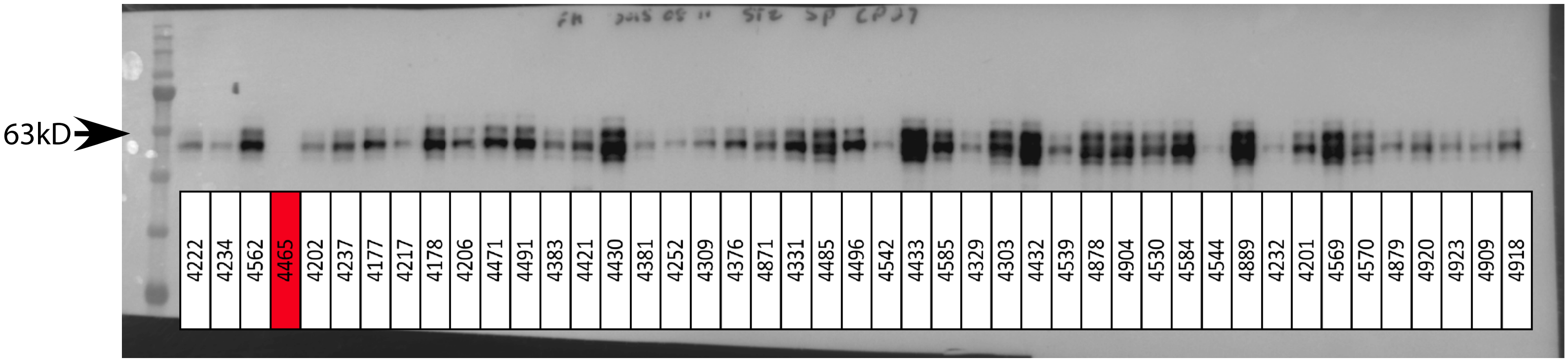

Figure 5

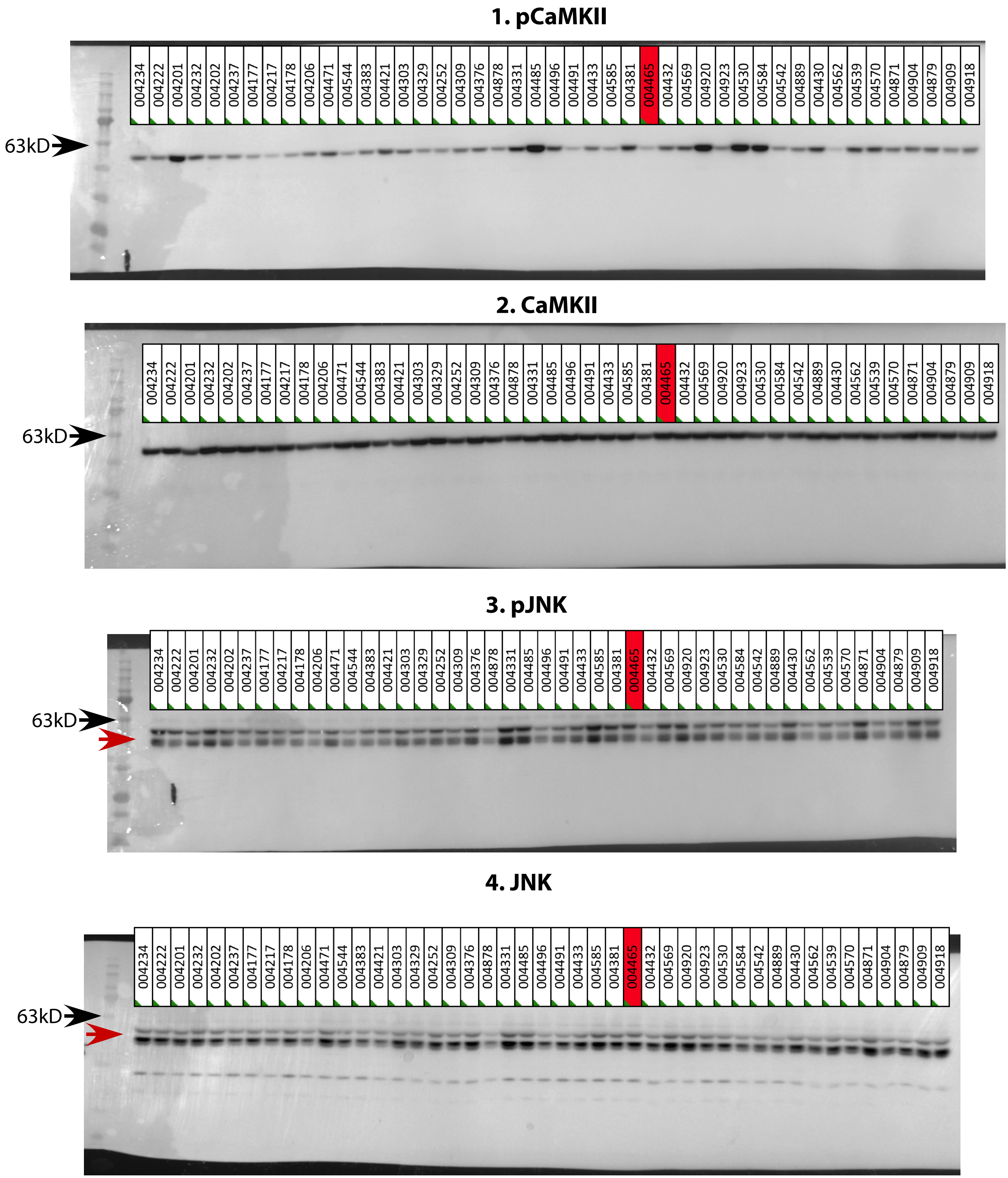
1. pCaMKII
63kD
004234 004222 004201 004232 004202 004237 004177 004217 004178 004206 004471 004544 004383 004421 004303 004329 004252 004309 004376 004878 004331 004485 004496 004491 004433 004585 004381 004465 004432 004569 004920 004923 004530 004584 004542 004889 004430 004562 004539 004570 004871 004904 004879 004909 004918
2. CaMKII
63kD
004234 004222 004201 004232 004202 004237 004177 004217 004178 004206 004471 004544 004383 004421 004303 004329 004252 004309 004376 004878 004331 004485 004496 004491 004433 004585 004381 004465 004432 004569 004920 004923 004530 004584 004542 004889 004430 004562 004539 004570 004871 004904 004879 004909 004918
3. pJNK
63kD
004234 004222 004201 004232 004202 004237 004177 004217 004178 004206 004471 004544 004383 004421 004303 004329 004252 004309 004376 004878 004331 004485 004496 004491 004433 004585 004381 004465 004432 004569 004920 004923 004530 004584 004542 004889 004430 004562 004539 004570 004871 004904 004879 004909 004918
4. JNK
63kD
004234 004222 004201 004232 004202 004237 004177 004217 004178 004206 004471 004544 004383 004421 004303 004329 004252 004309 004376 004878 004331 004485 004496 004491 004433 004585 004381 004465 004432 004569 004920 004923 004530 004584 004542 004889 004430 004562 004539 004570 004871 004904 004879 004909 004918

5. pS9-GSK3β

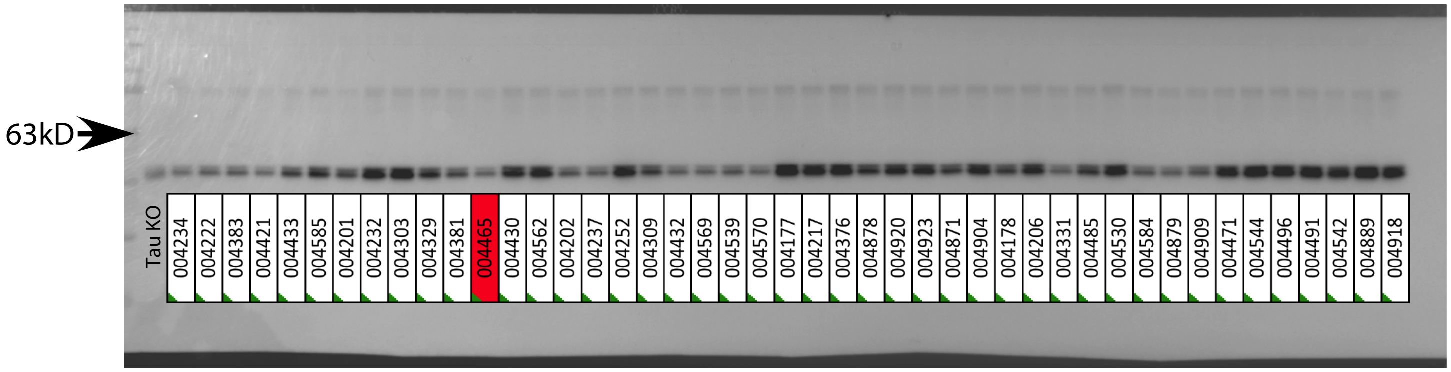

6. GSK3β

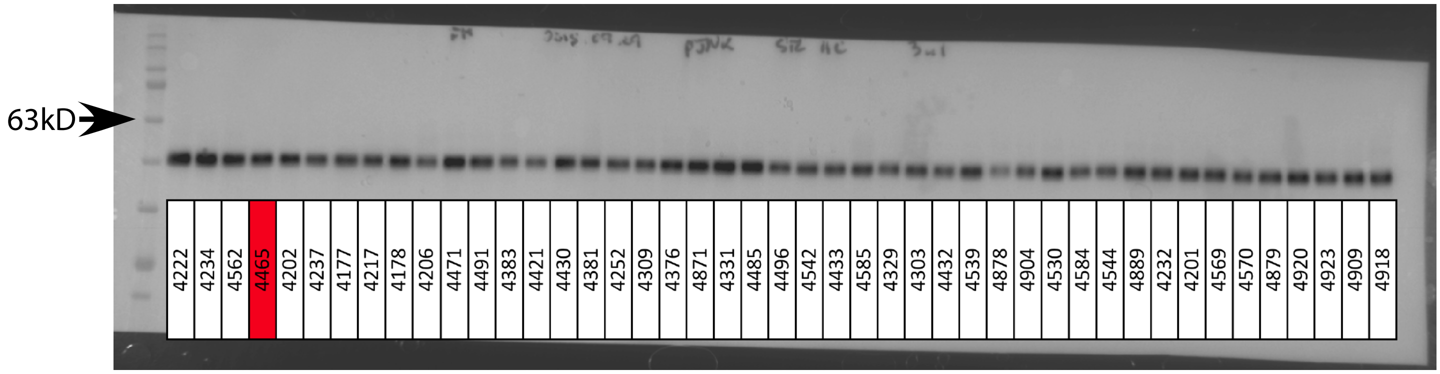

7. pP38

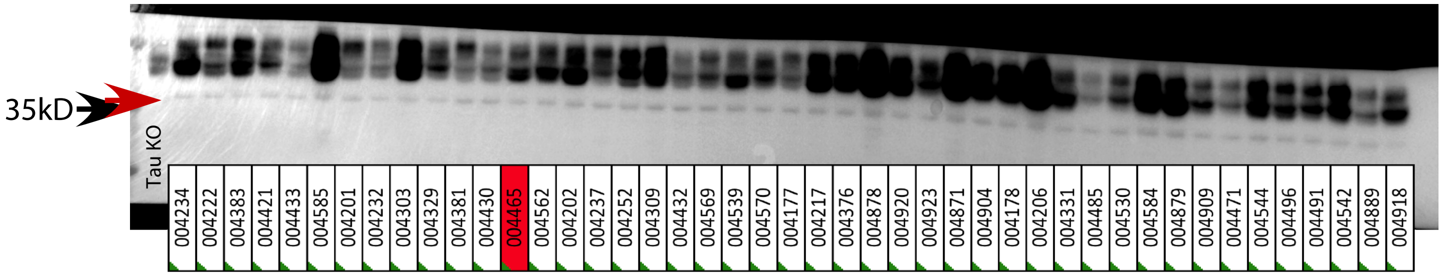

8. P38

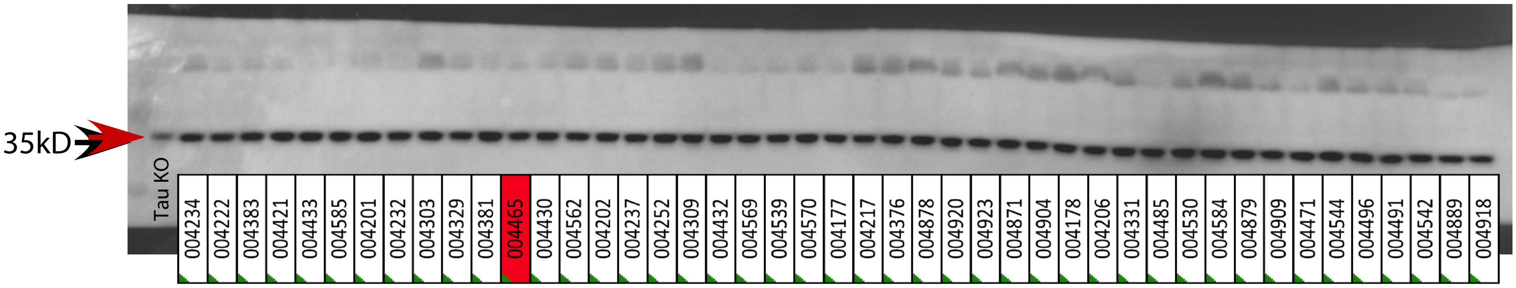

9. pERK

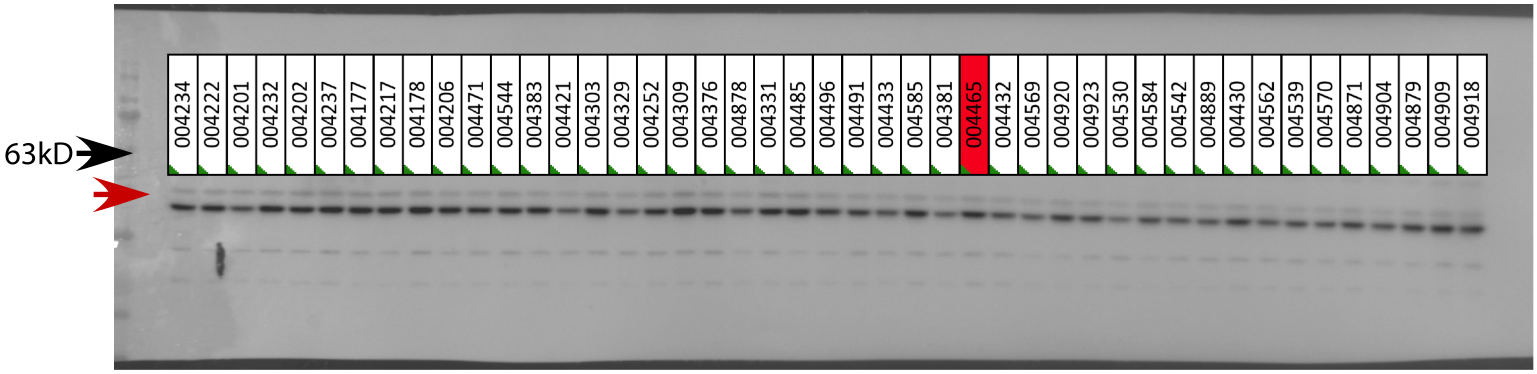

10. ERK

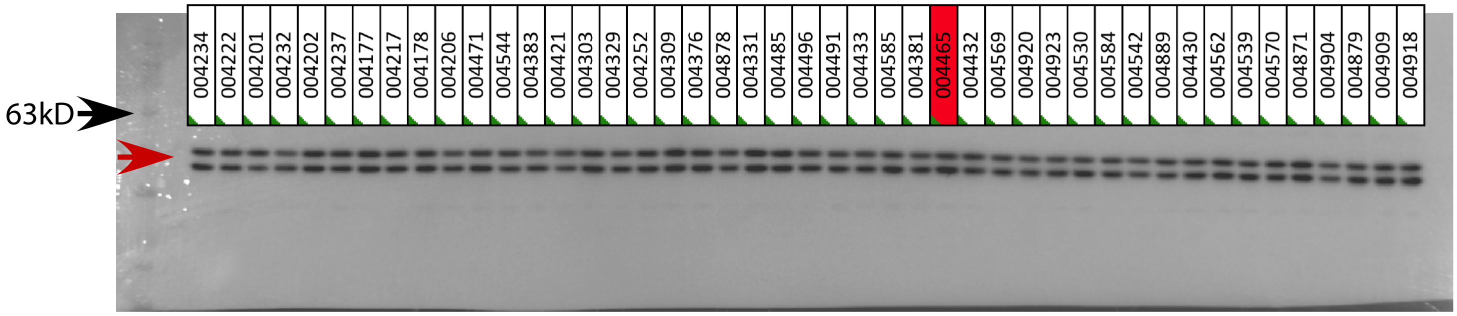

11. cdk5

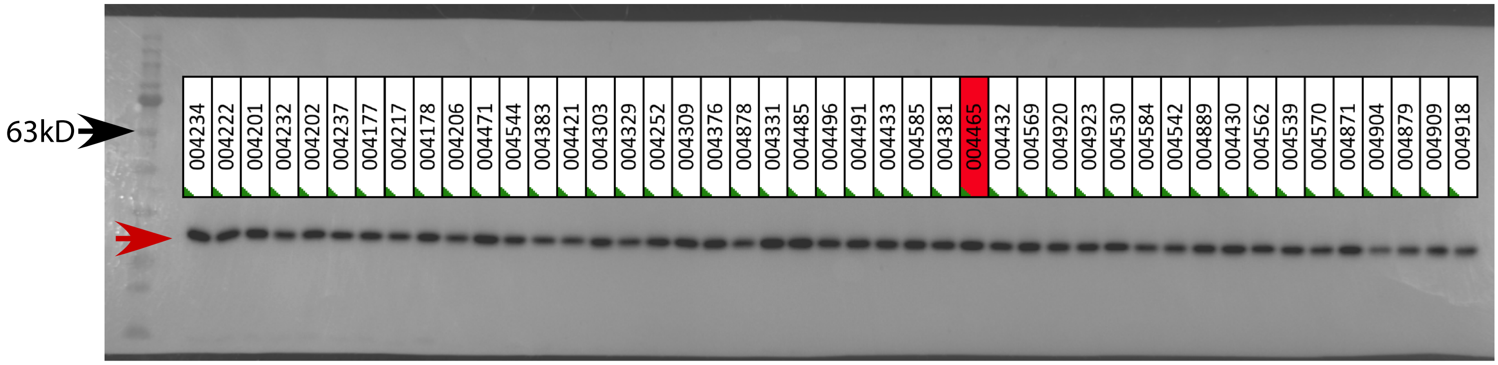

12. p35

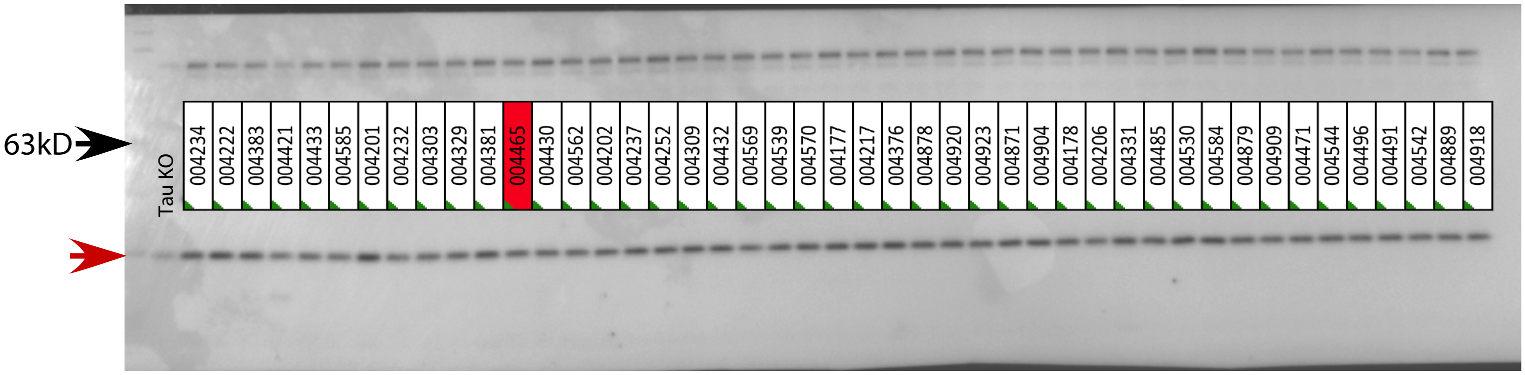

Figure 6

1. PP1

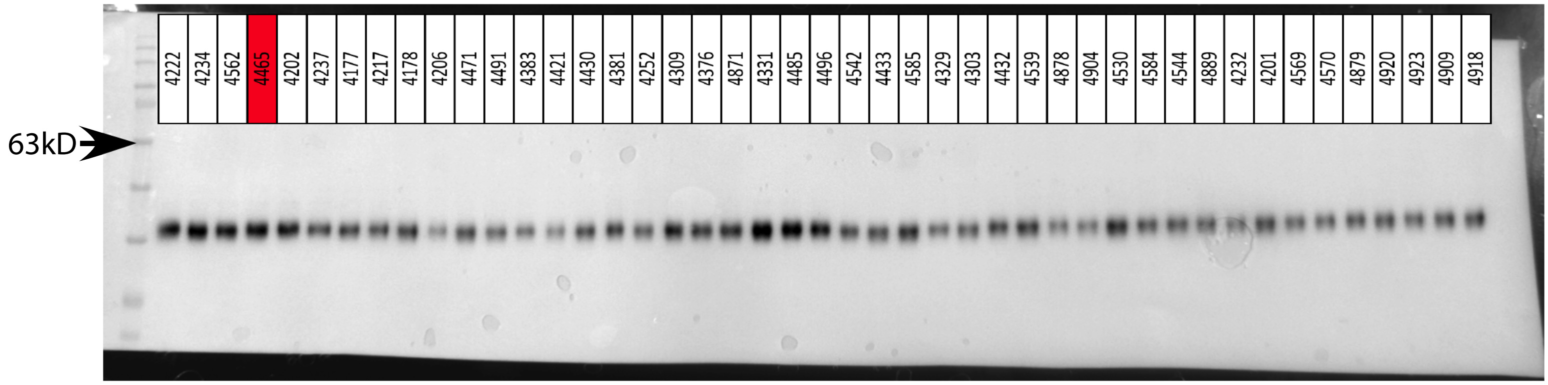

2. PP2A-A

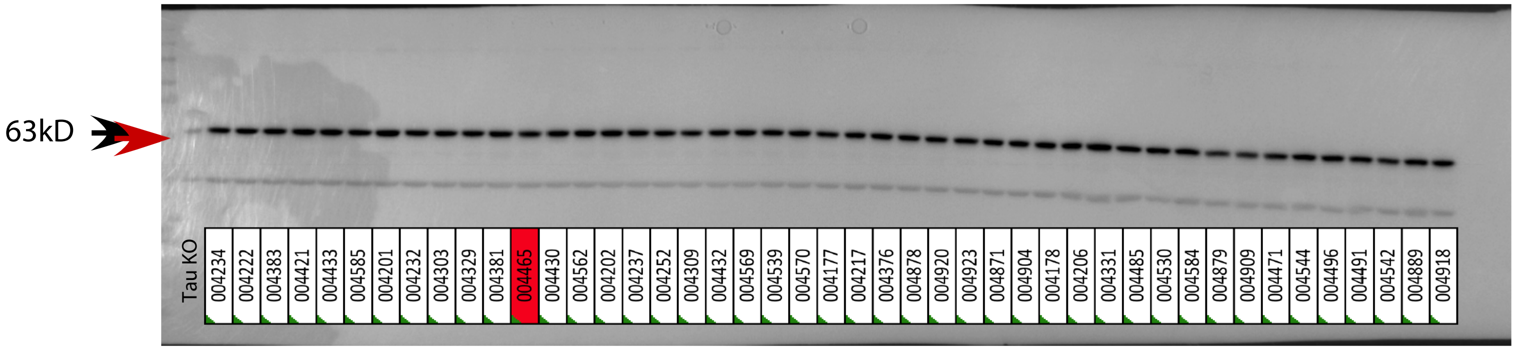

3. PP2A-Bα

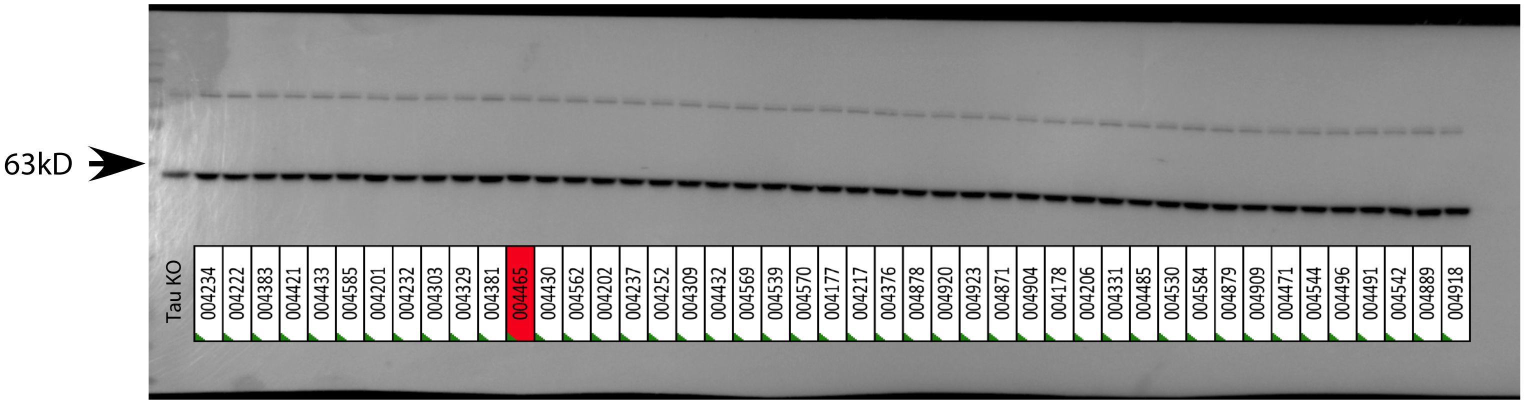

4. PP2A-Bβ

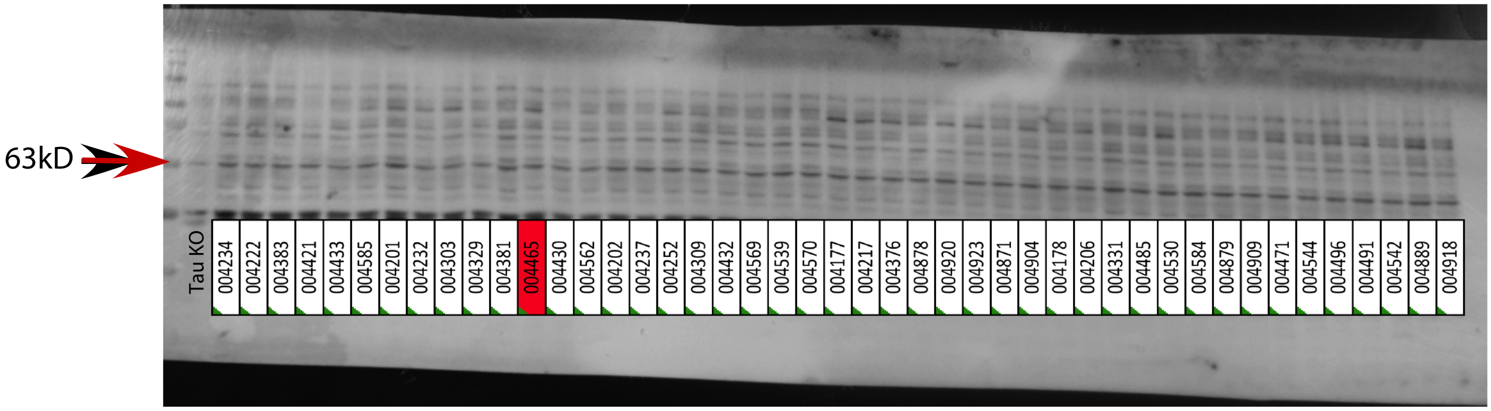

5. demPP2A-C

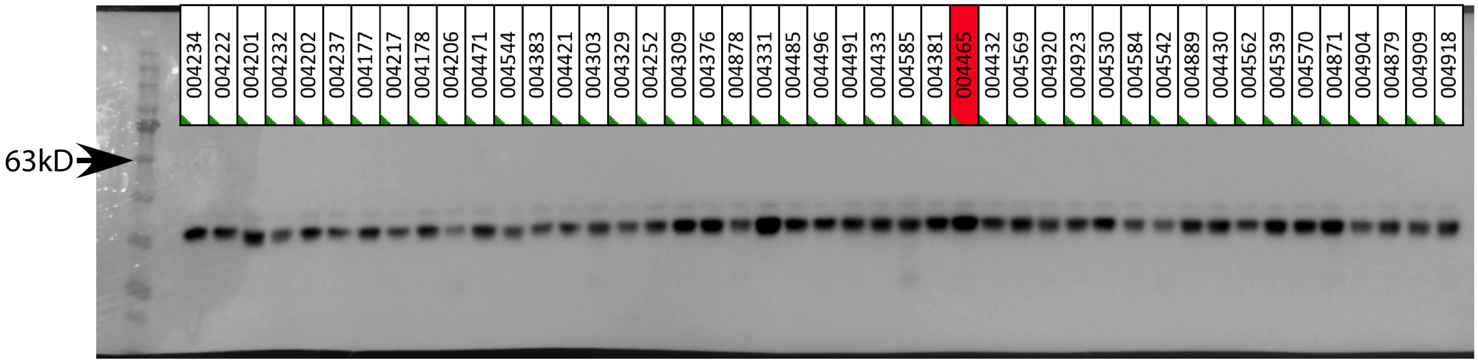

6. PP2A-C

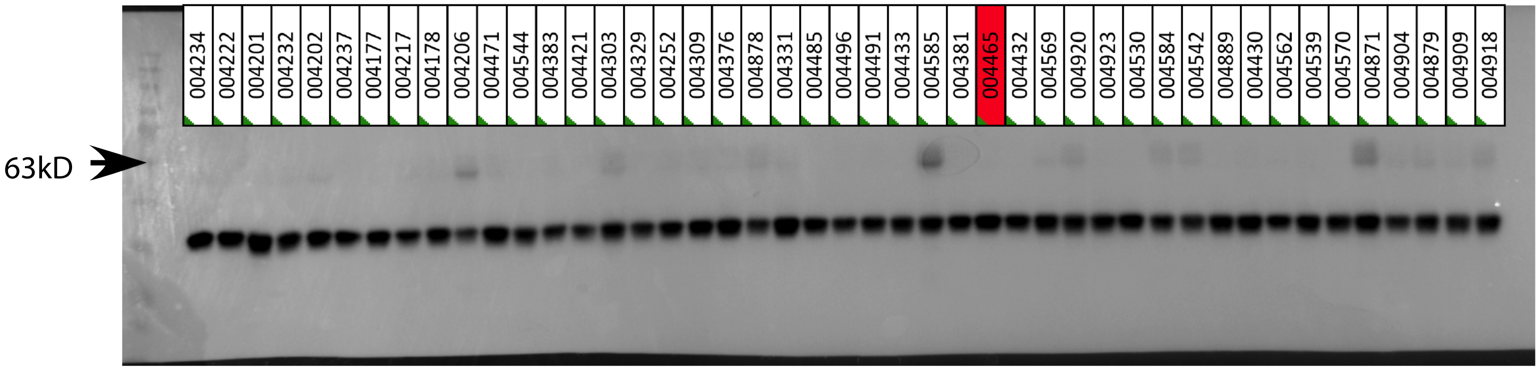

7. PP2B

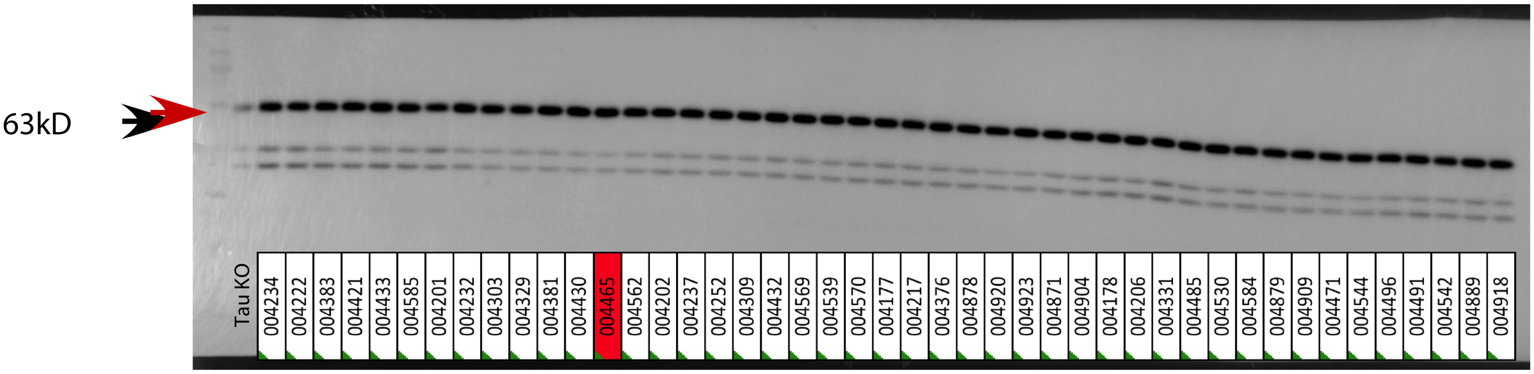

8. PP5

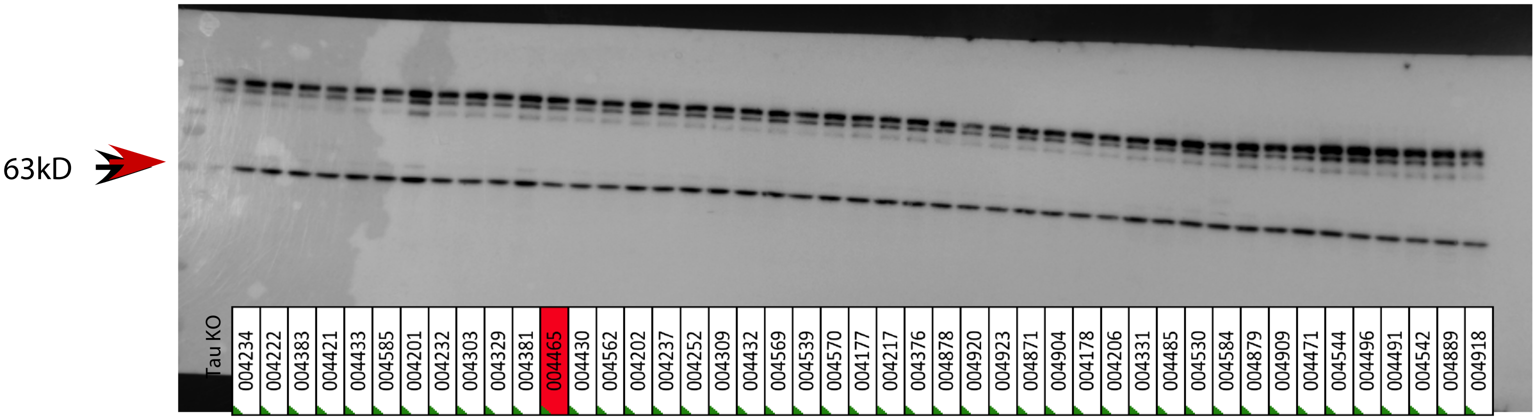

9. pPTEN

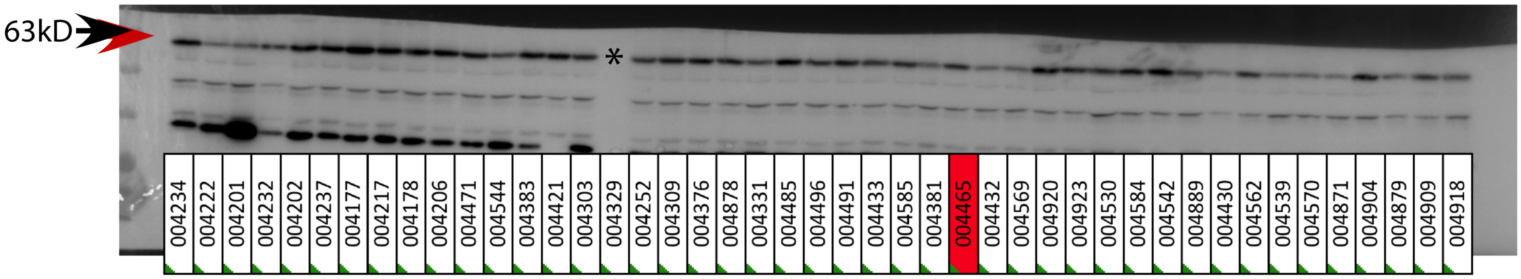

10. PTEN

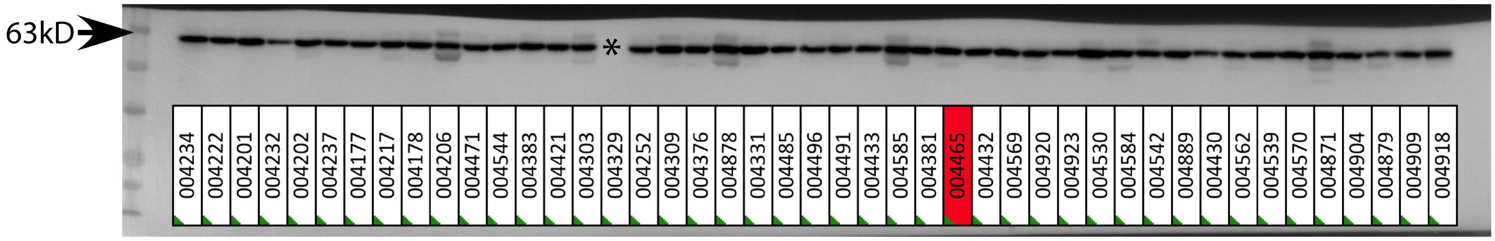

* No more sample

Figure 7

1. pIR

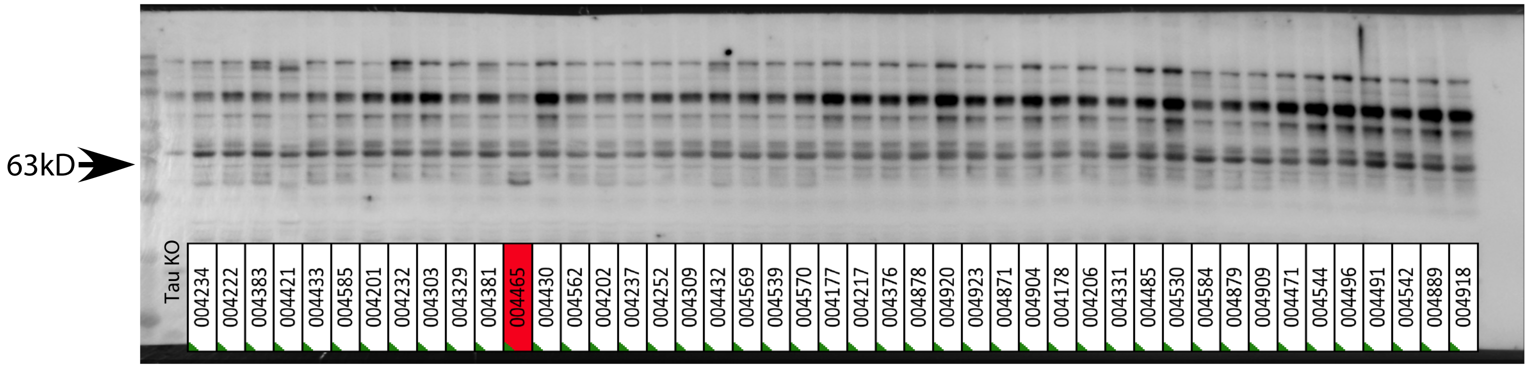

2. IR

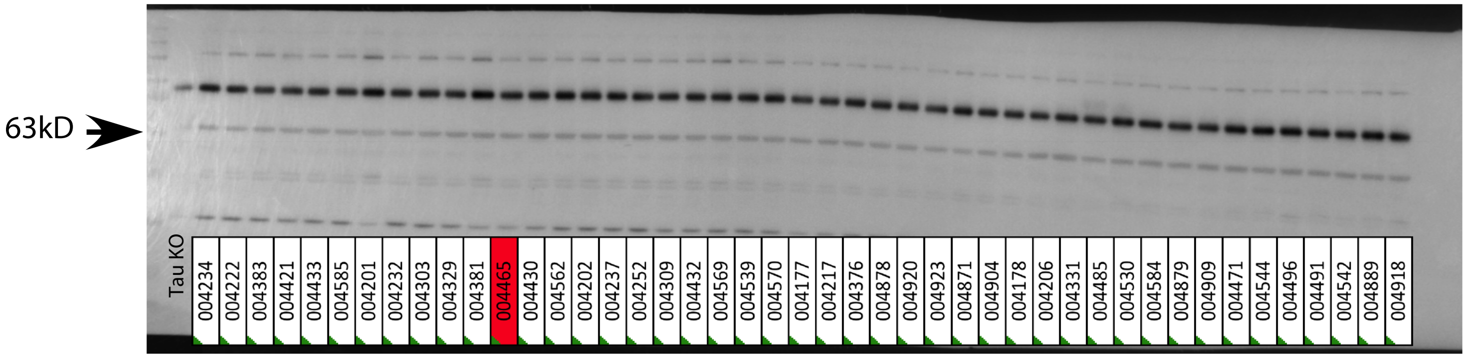

3. pIGF-1 R

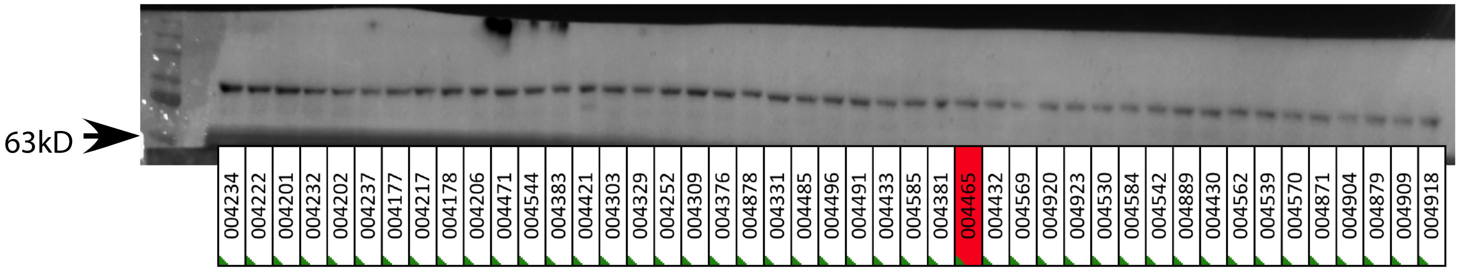

4. IGF-1 R

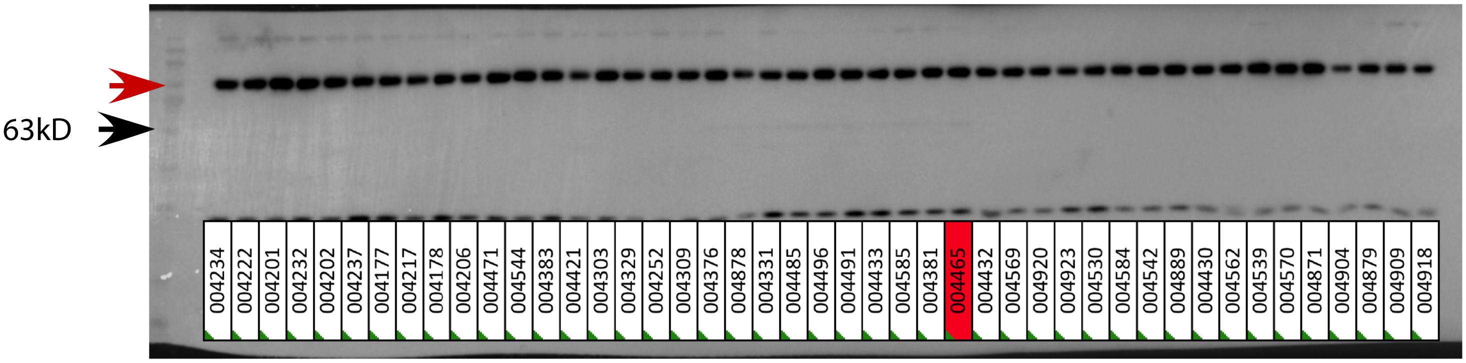

5. pPI3K

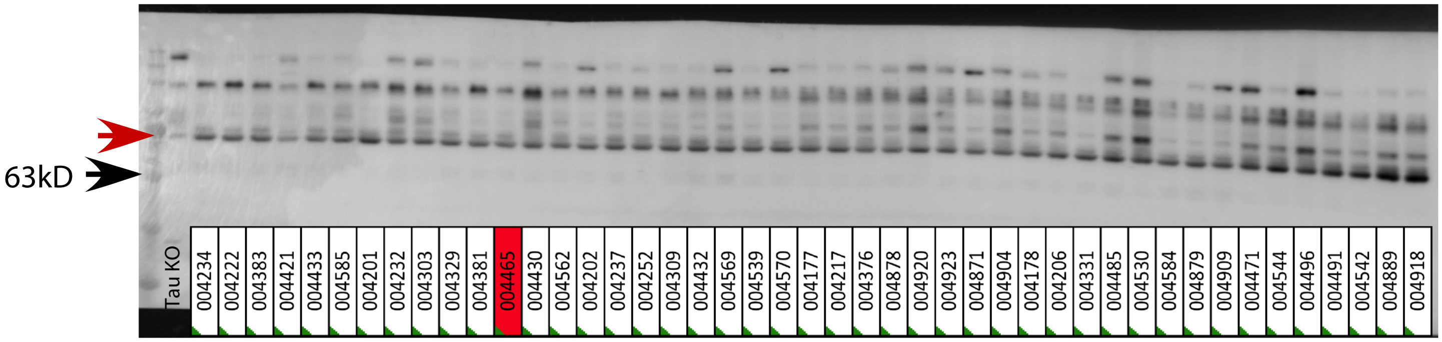

6. PI3K

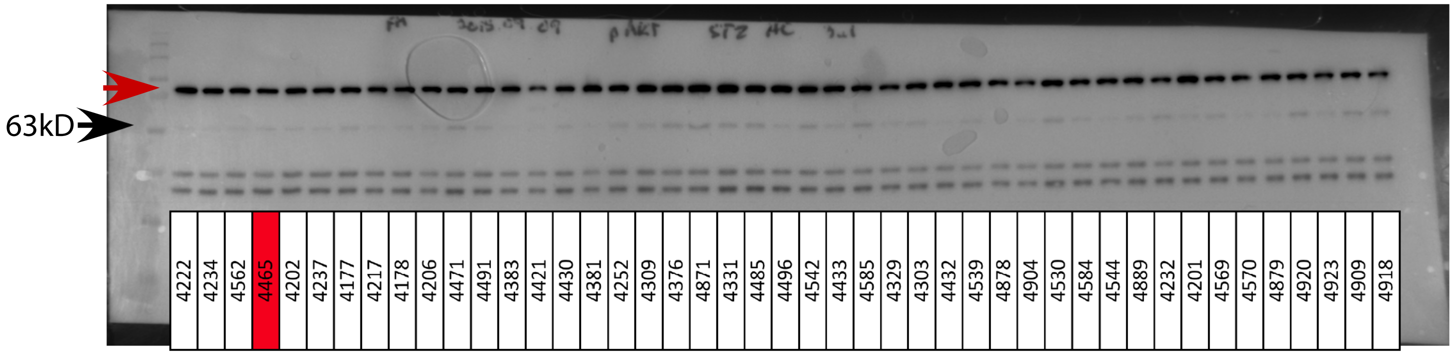

7. pAKT

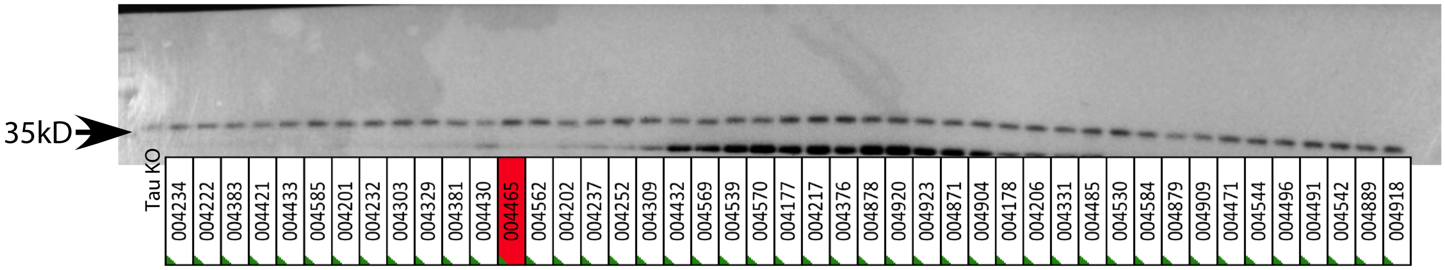

8. AKT

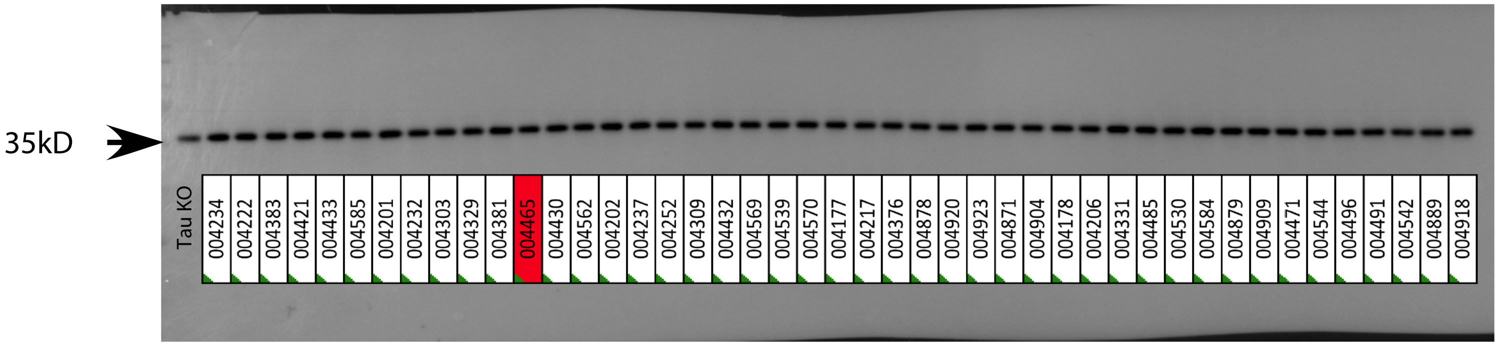

Figure 8

1. Drebrin

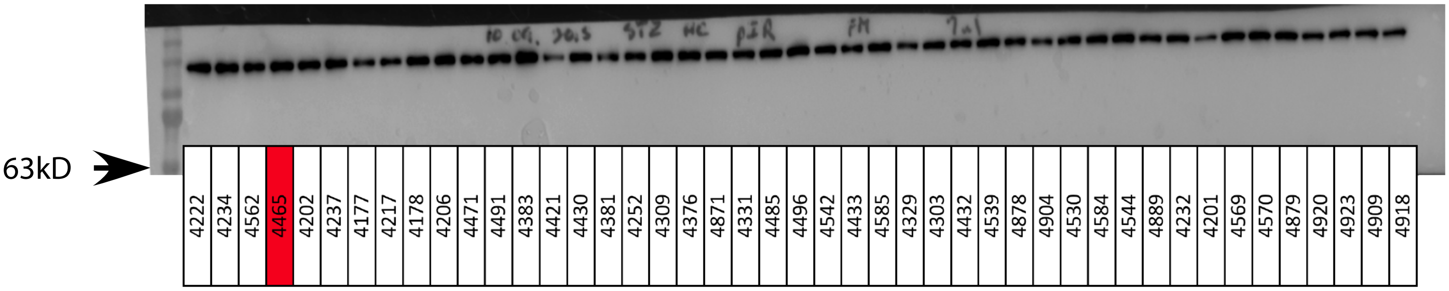

2. Synaptophysin

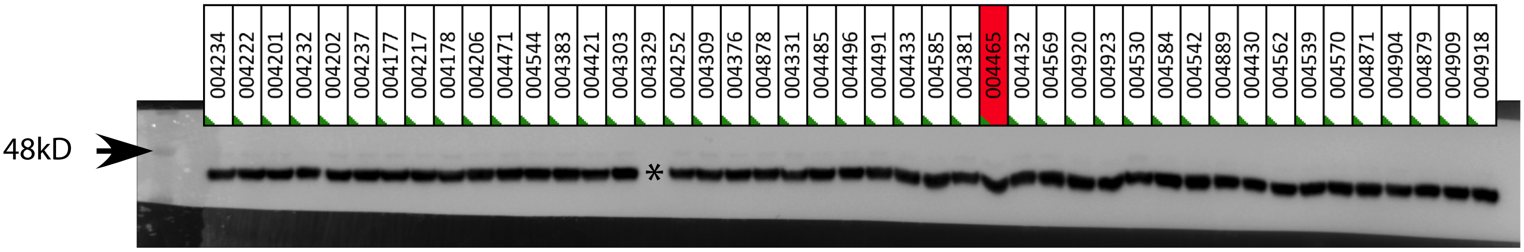

3. SNAP25

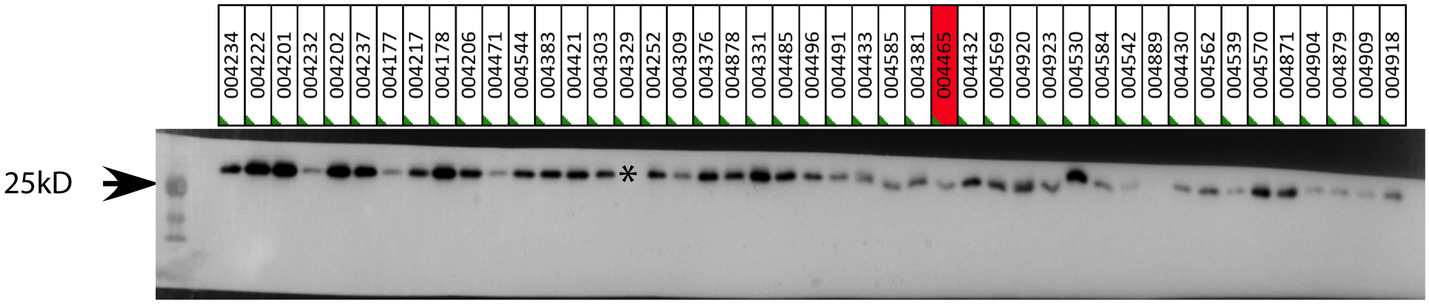

4. PSD95

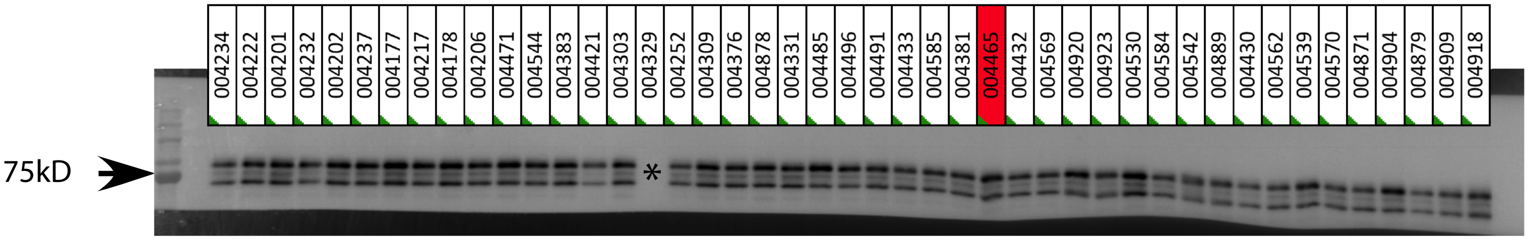

* No more sample
